# Supplementary material for: Glutaredoxin catalysis requires two distinct glutathione interaction sites
Source: Nat Commun. 2017 Apr 4;8:14835. doi: 10.1038/ncomms14835 (PMC5382279; doi:10.1038/ncomms14835)
Supplement: Supplementary Information — Supplementary Figures, Supplementary Tables. [file ncomms14835-s1.pdf]

Supplementary Results

Supplementary Figure 1

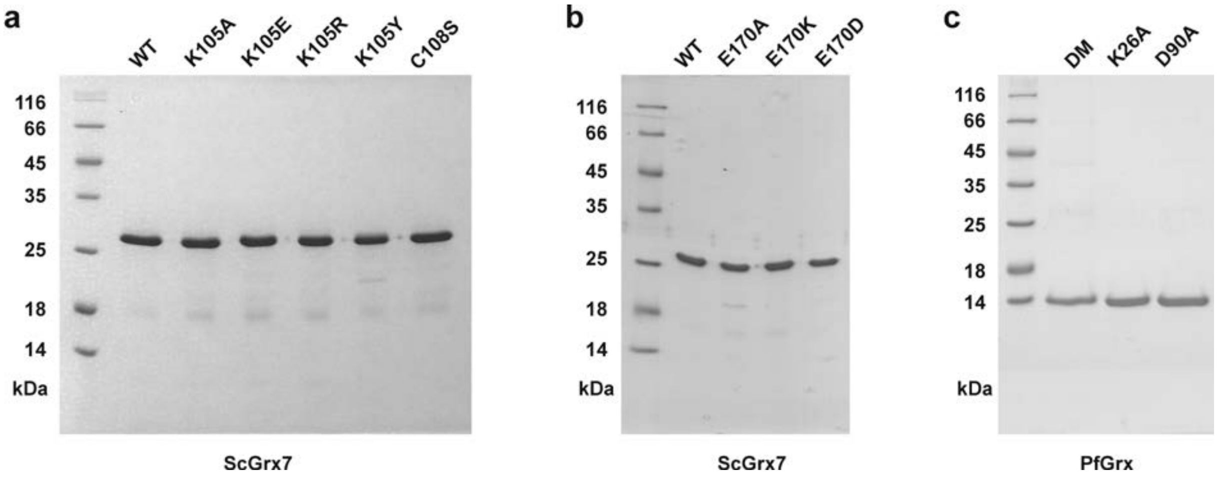

Supplementary Figure 1 | Purification of recombinant wild type and mutant enzymes. SDS-PAGE analysis of representative eluates after protein purification by Ni-NTA affinity chromatography. (**a**) 15% gel of Lys105 mutants of ScGrx7. ScGrx7 wild type enzyme, WT; ScGrx7^{K105A}, K105A; ScGrx7^{K105E}, K105E; ScGrx7^{K105R}, K105R; ScGrx7^{K105Y}, K105Y; ScGrx7^{C108S}, C108S. ScGrx7^{C108S} served as a negative control and was inactive in all assays. (**b**) 15% gel of Glu170 mutants of ScGrx7. ScGrx7 wild type enzyme, WT; ScGrx7^{E170A}, E170A; ScGrx7^{E170K}, E170K; ScGrx7^{E170D}, E170D. Average yields from up to nine independent protein purification experiments for each mutant were highly reproducible and ranged from 6.2±1.1 to 7.8±1.2 mg of recombinant ScGrx7 per liter of *E. coli* culture. (**c**) 15% gel of PfGrx mutants. Double cysteine mutant PfGrx$^{C32S/C88S}$, DM; PfGrx$^{K26A/C32S/C88S}$, K26A; PfGrx$^{C32S/C88S/D90A}$, D90A. Average yields from up to seven independent protein purification experiments were 8.8±3.8 mg per liter of *E. coli* culture for DM, 31±10 mg for K26A and 5.1±1.9 mg for D90A .

Supplementary Figure 2

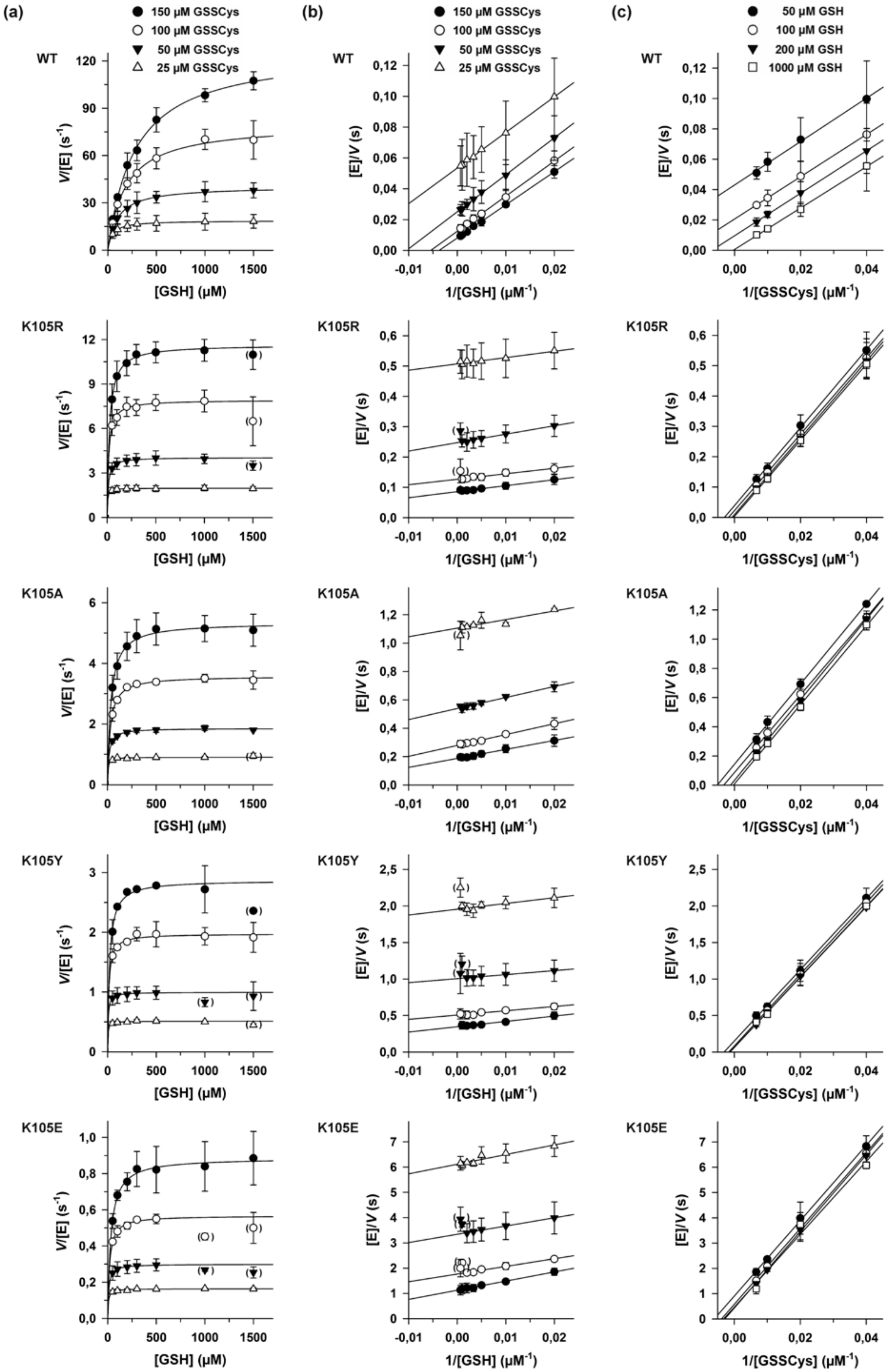

Supplementary Figure 2 | GSSCys assay steady-state kinetics of ScGrx7 wild type enzyme and K105X mutants. (**a**) Michaelis-Menten plots of the GSH-dependent reaction velocity at different initial concentrations of GSSCys. (**b**) Lineweaver-Burk plots of the GSH-dependent reaction velocity at different initial concentrations of GSSCys revealing ping-pong kinetic patterns. (**c**) Lineweaver-Burk plots of the GSSCys-dependent reaction velocity at different initial concentrations of GSH. All data points are the mean ± S.D. from at least three independent replicates and were averaged from at least two independent protein purification experiments and subsequently plotted and fitted in Sigmaplot 12.5 according to Michaelis-Menten, Lineweaver-Burk, Eadie-Hofstee and Hanes theory (the latter two plots are not shown). Calculated k_{cat}^{app} and K_m^{app} values from the four different plots usually varied by less than 10%. Data points in brackets were omitted from the regression analysis when the k_{cat}^{app} or K_m^{app} values from all four plots varied by more than 10% and converged after removal of the outlier. Estimated true kinetic constants are listed in Supplementary Table 1. Selected apparent kinetic constants from non-linear regression analyses of Michaelis-Menten plots are listed in Supplementary Table 2.

Supplementary Figure 3

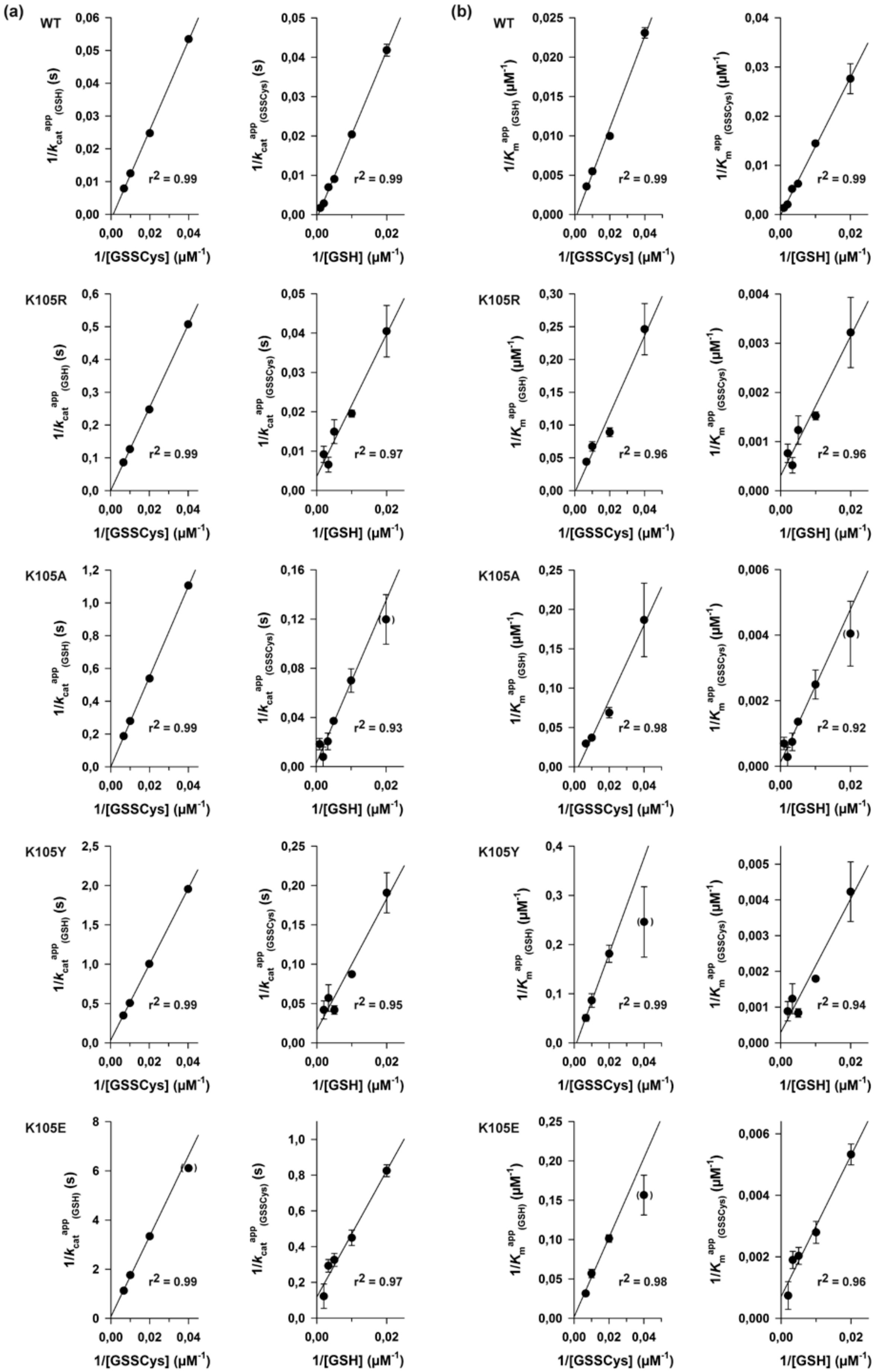

Supplementary Figure 3 | GSSCys assay secondary plots for ScGrx7 wild type enzyme and K105X mutants. (**a**) Secondary plots of the k_{cat}^{app} values at different concentrations of GSSCys (left panels) and GSH (right panels) allowing the estimation of the true k_{cat} value from the y-axis intercept ($1/k_{cat}$) and of the true K_m value from the x-axis intercept ($-1/K_m$). (**b**) Secondary plots of the K_m^{app} values at different concentrations of GSSCys (left panels) and GSH (right panels). K_m^{app} and k_{cat}^{app} values were obtained from non-linear regression analyses of Michaelis-Menten plots (Supplementary Fig. 2). Outliers in brackets at the lowest substrate concentration were identified based on the r^2 values and omitted from the linear regression analysis in Sigmaplot 12.5. Estimated true kinetic constants and Dalziel coefficients are listed in Supplementary Table 1.

Supplementary Figure 4

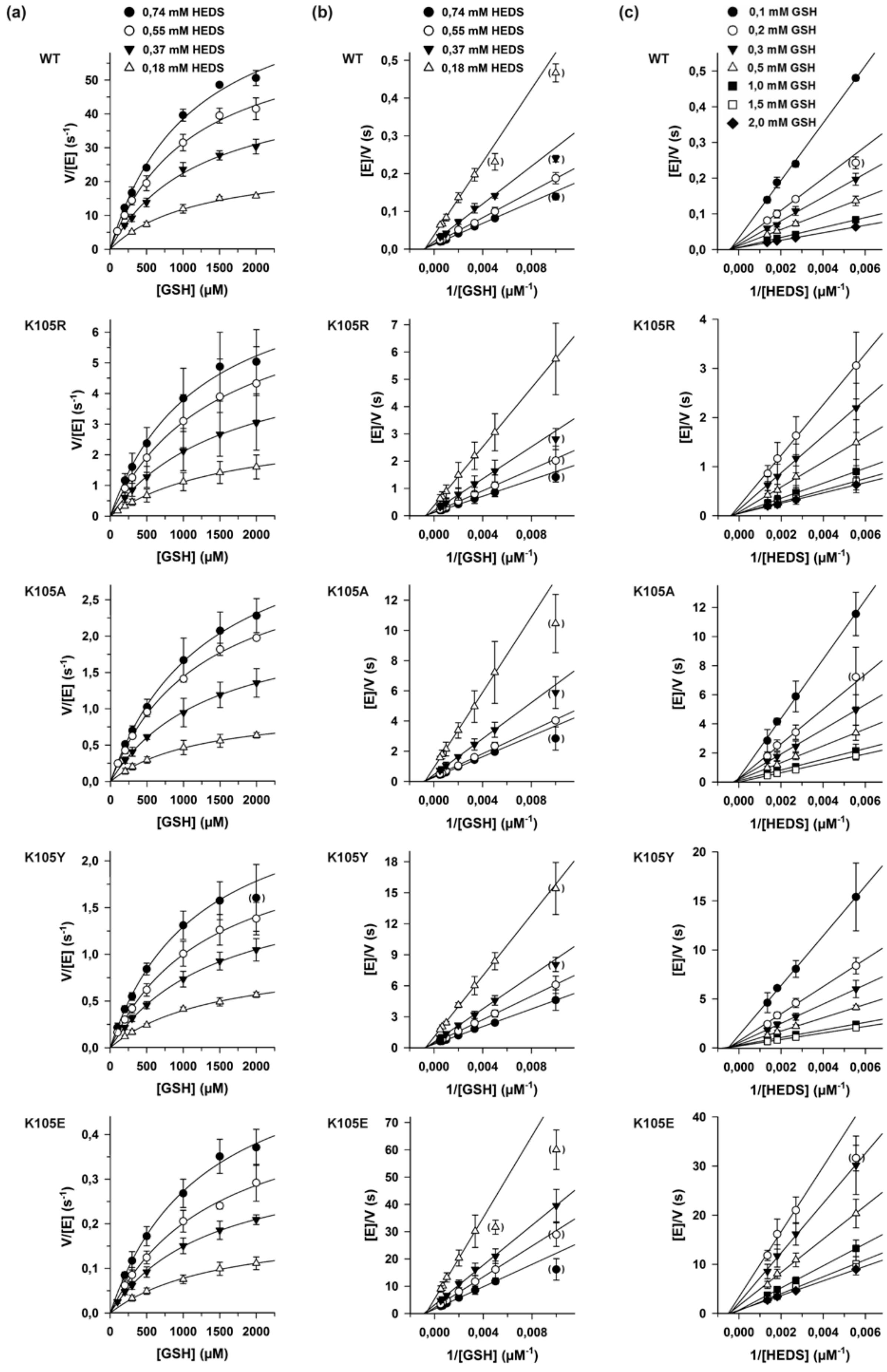

Supplementary Figure 4 | HEDS assay steady-state kinetics of ScGrx7 wild type enzyme and K105X mutants. (**a**) Michaelis-Menten plots of the GSH-dependent reaction velocity at different initial concentrations of HEDS. (**b**) Lineweaver-Burk plots of the GSH-dependent reaction velocity at different initial concentrations of HEDS revealing sequential kinetic patterns. (**c**) Lineweaver-Burk plots of the HEDS-dependent reaction velocity at different initial concentrations of GSH. All data points are the mean ± S.D. from at least three independent replicates and were averaged from at least three independent protein purification experiments and subsequently plotted and fitted in Sigmaplot 12.5 according to Michaelis-Menten, Lineweaver-Burk, Eadie-Hofstee and Hanes theory (the latter two plots are not shown). Calculated k_{cat}^{app} and K_m^{app} values from the four different plots usually varied by less than 10%. Data points in brackets were omitted from the regression analysis when the k_{cat}^{app} or K_m^{app} values from all four plots varied by more than 10% and converged after removal of the outlier. Estimated true kinetic constants are listed in Supplementary Table 1. Selected apparent kinetic constants from non-linear regression analyses of Michaelis-Menten plots are listed in Supplementary Table 3.

Supplementary Figure 5

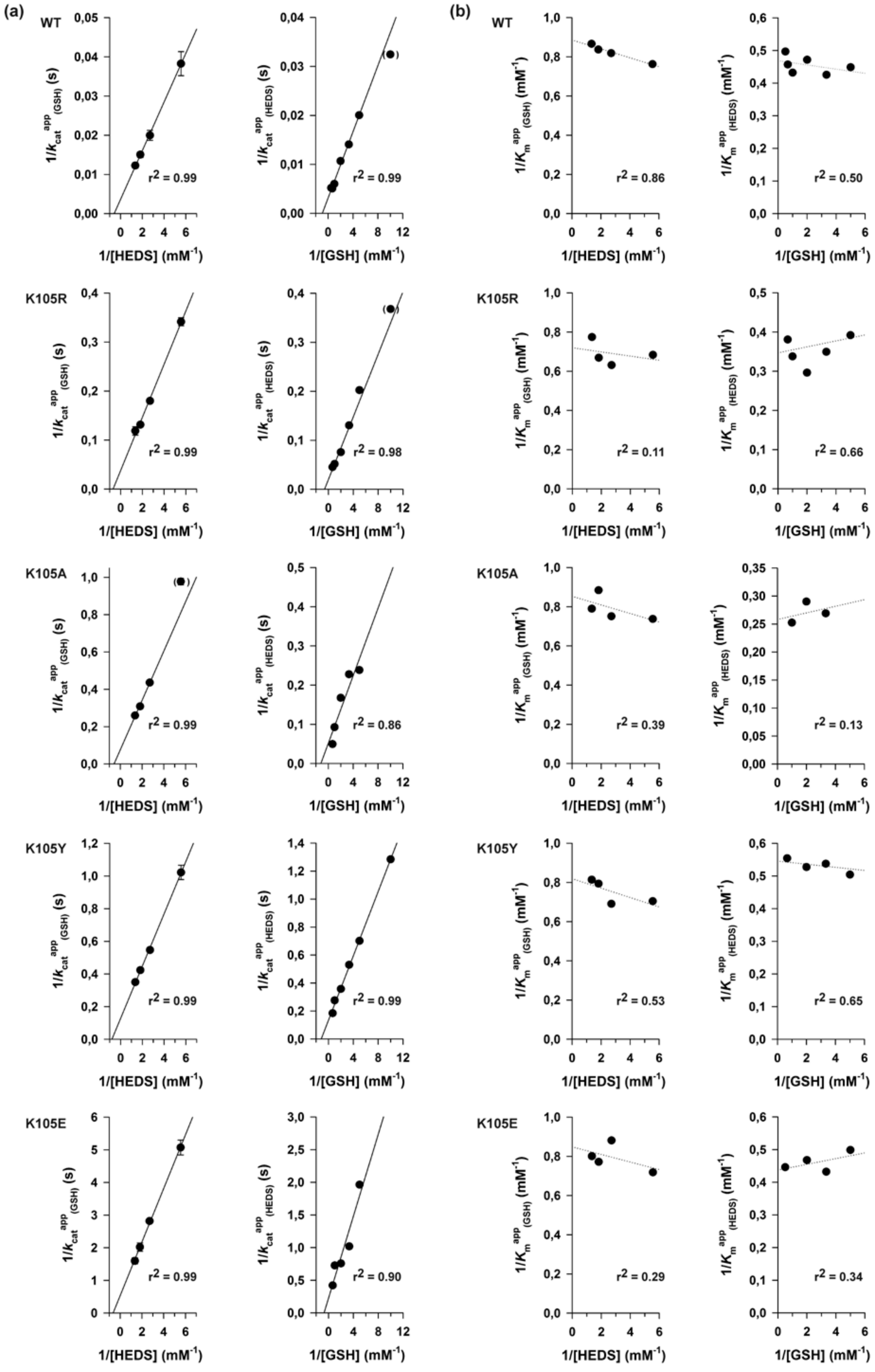

Supplementary Figure 5 | HEDS assay secondary plots for ScGrx7 wild type enzyme and K105X mutants. K_m^{app} and k_{cat}^{app} values were obtained from non-linear regression analyses of Michaelis-Menten plots (Supplementary Fig. 4). (**a**) Secondary plots of the k_{cat}^{app} values at different concentrations of HEDS (left panels) and GSH (right panels) allowing the estimation of the true k_{cat} value from the y-axis intercept ($1/k_{cat}$) and of the true K_m value from the x-axis intercept ($-1/K_m$). Outliers in brackets at the lowest substrate concentration were identified based on the r^2 values and omitted from the linear regression analysis in Sigmaplot 12.5. (**b**) Secondary plots of the K_m^{app} values at different concentrations of HEDS (left panels) and GSH (right panels). Please note that the differences for K_m^{app} values among independent measurements and mutants are not statistically significant (see also Supplementary Table 11). Estimated true kinetic constants and Dalziel coefficients are listed in Supplementary Table 1.

Supplementary Figure 6

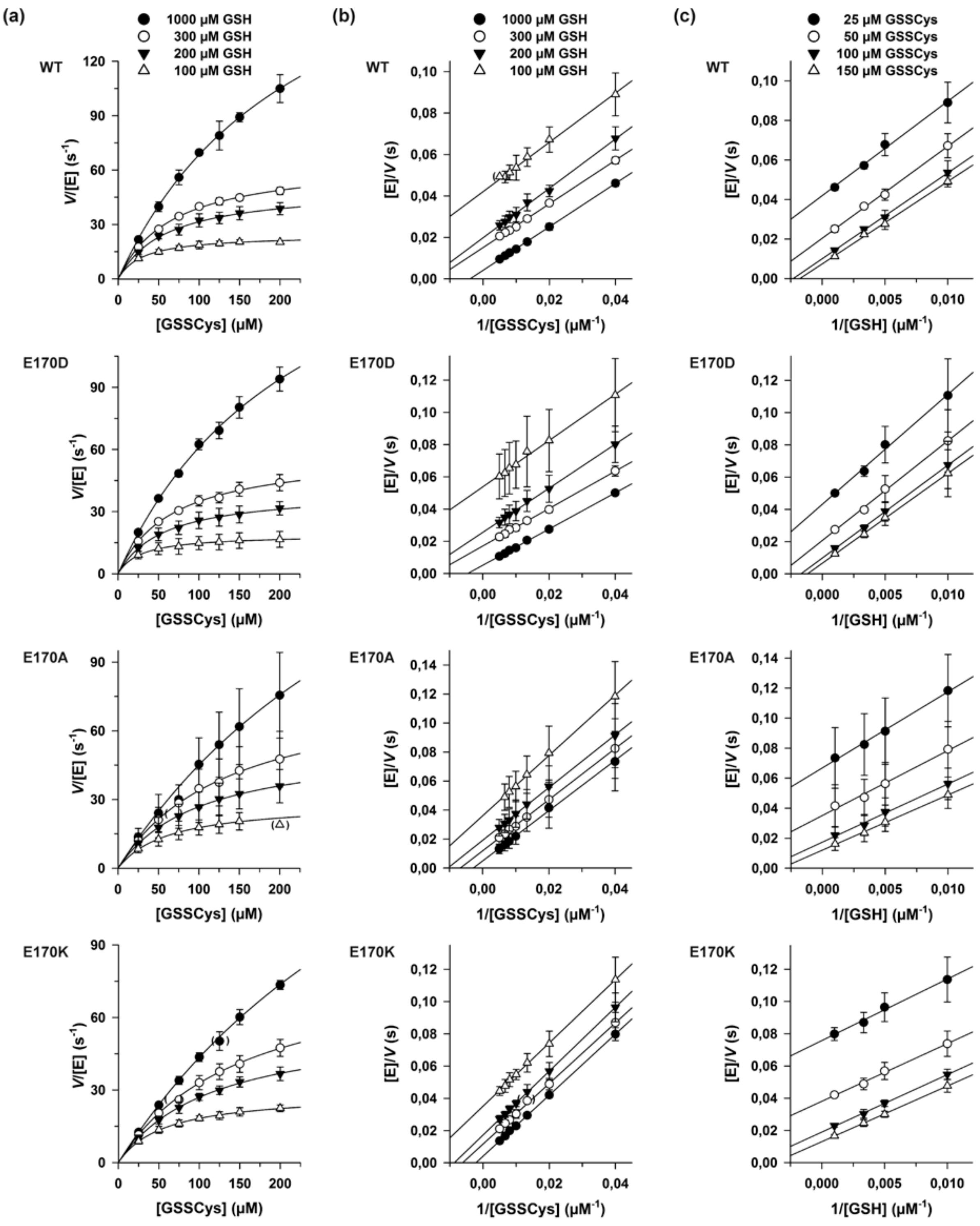

Supplementary Figure 6 | GSSCys assay steady-state kinetics of ScGrx7 wild type enzyme and E170X mutants. (**a**) Michaelis-Menten plots of the GSH-dependent reaction velocity at different initial concentrations of GSSCys. (**b**) Lineweaver-Burk plots of the GSH-dependent reaction velocity at different initial concentrations of GSSCys revealing ping-pong kinetic patterns. (**c**) Lineweaver-Burk plots of the GSSCys-dependent reaction velocity at different initial concentrations of GSH. All data points are the mean ± S.D. from at least three independent replicates and were averaged from at least two independent protein purification experiments and subsequently plotted and fitted in Sigmaplot 12.5 according to Michaelis-Menten, Lineweaver-Burk, Eadie-Hofstee and Hanes theory (the latter two plots are not shown). Calculated k_{cat}^{app} and K_m^{app} values from the four different plots usually varied by less than 10%. Data points in brackets were omitted from the regression analysis when the k_{cat}^{app} or K_m^{app} values from all four plots varied by more than 10% and converged after removal of the outlier. Estimated true kinetic constants are listed in Supplementary Table 1. Selected apparent kinetic constants from non-linear regression analyses of Michaelis-Menten plots are listed in Supplementary Table 4.

Supplementary Figure 7

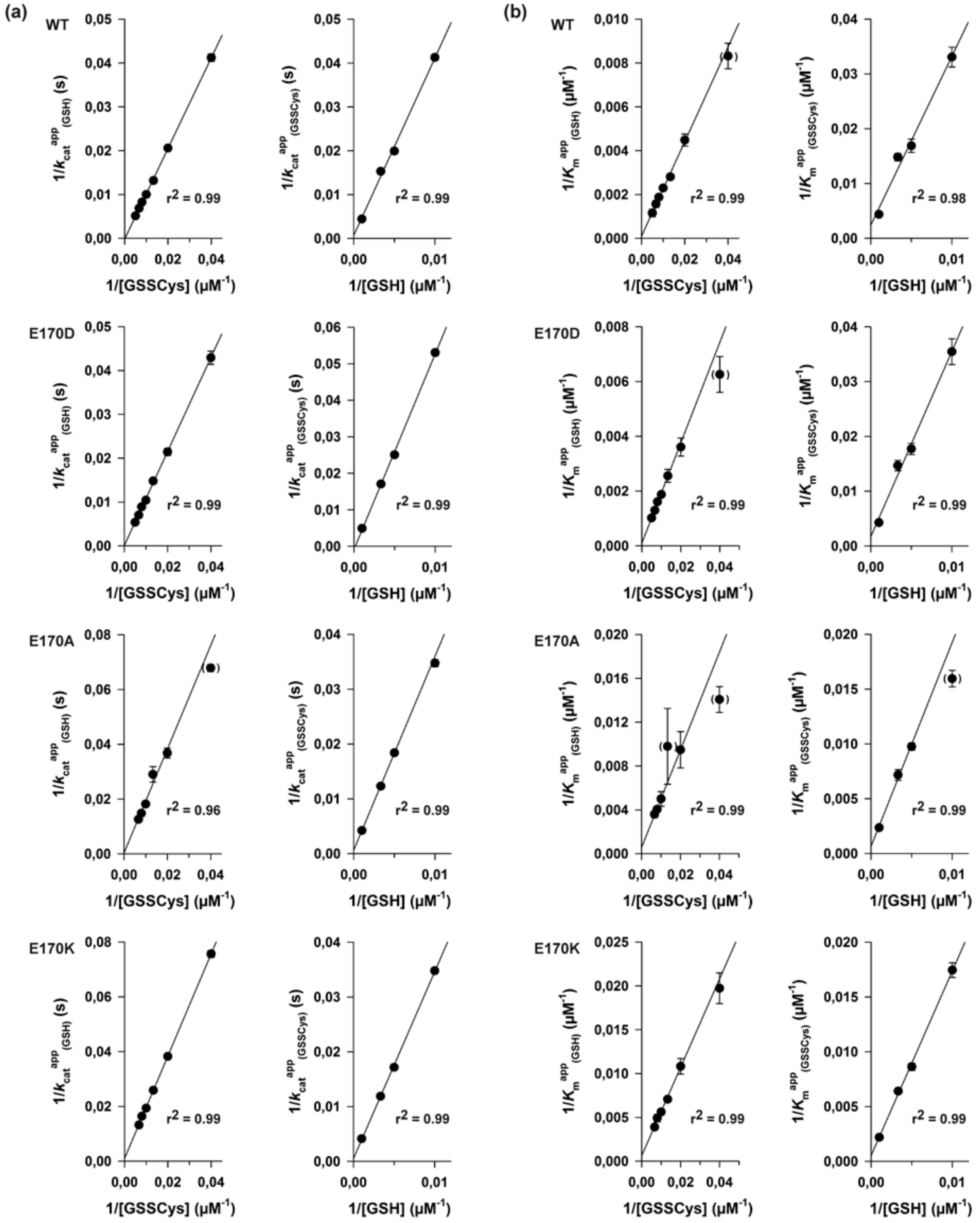

Supplementary Figure 7 | GSSCys assay secondary plots for ScGrx7 wild type enzyme and E170X mutants. (**a**) Secondary plots of the k_{cat}^{app} values at different concentrations of GSSCys (left panels) and GSH (right panels) allowing the estimation of the true k_{cat} value from the y-axis intercept ($1/k_{cat}$) and of the true K_m value from the x-axis intercept ($-1/K_m$). (**b**) Secondary plots of the K_m^{app} values at different concentrations of GSSCys (left panels) and GSH (right panels). K_m^{app} and k_{cat}^{app} values were obtained from non-linear regression analyses of Michaelis-Menten plots (Supplementary Fig. 6). Outliers in brackets at the lowest substrate concentration were identified based on the r^2 values and omitted from the linear regression analysis in Sigmaplot 12.5. Estimated true kinetic constants and Dalziel coefficients are listed in Supplementary Table 1.

Supplementary Figure 8

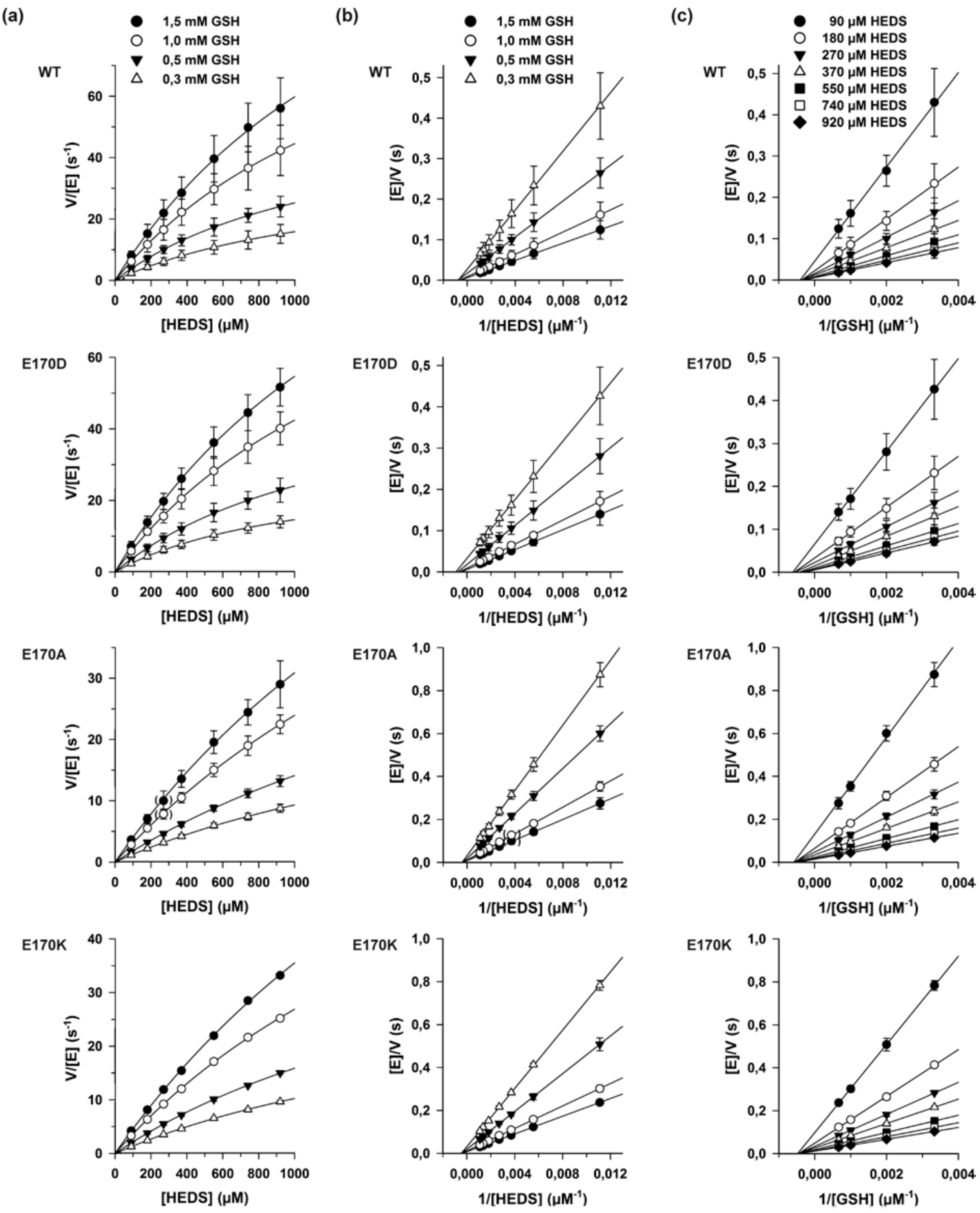

Supplementary Figure 8 | HEDS assay steady-state kinetics of ScGrx7 wild type enzyme and E170X mutants. (**a**) Michaelis-Menten plots of the HEDS-dependent reaction velocity at different initial concentrations of GSH. (**b**) Lineweaver-Burk plots of the HEDS-dependent reaction velocity revealing sequential kinetic patterns. (**c**) Lineweaver-Burk plots of the GSH-dependent reaction velocity. All data points are the mean ± S.D. from at least three independent replicates and were averaged from at least three independent protein purification experiments and subsequently plotted and fitted in Sigmaplot 12.5 according to Michaelis-Menten, Lineweaver-Burk, Eadie-Hofstee and Hanes theory (the latter two plots are not shown). Calculated k_{cat}^{app} and K_m^{app} values from the four different plots usually varied by less than 10%. Data points in brackets were omitted from the regression analysis when the k_{cat}^{app} or K_m^{app} values from all four plots varied by more than 10% and converged after removal of the outlier. Estimated true kinetic constants are listed in Supplementary Table 1. Selected apparent kinetic constants from non-linear regression analyses of Michaelis-Menten plots are listed in Supplementary Table 5.

Supplementary Figure 9

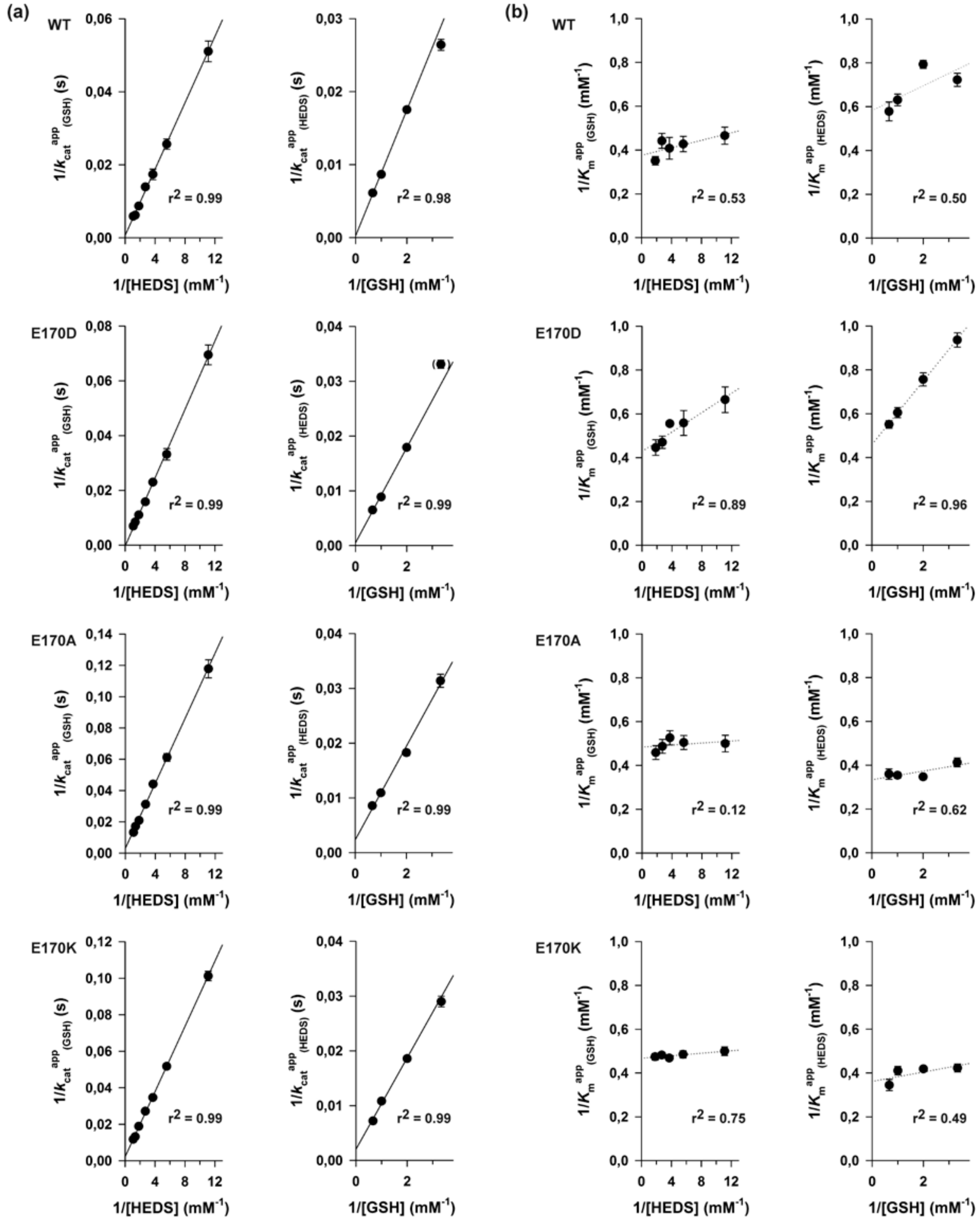

Supplementary Figure 9 | HEDS assay secondary plots for ScGrx7 wild type enzyme and E170X mutants. K_m^{app} and k_{cat}^{app} values were obtained from non-linear regression analyses of Michaelis-Menten plots (Supplementary Fig. 8). (**a**) Secondary plots of the k_{cat}^{app} values at different concentrations of HEDS (left panels) and GSH (right panels) allowing the estimation of the true k_{cat} value from the y-axis intercept ($1/k_{cat}$) and of the true K_m value from the x-axis intercept ($-1/K_m$). The outlier in brackets at the lowest substrate concentration was identified based on the r^2 value and omitted from the linear regression analysis in Sigmaplot 12.5. (**b**) Secondary plots of the K_m^{app} values at different concentrations of HEDS (left panels) and GSH (right panels). Estimated true kinetic constants and Dalziel coefficients are listed in Supplementary Table 1.

Supplementary Figure 10

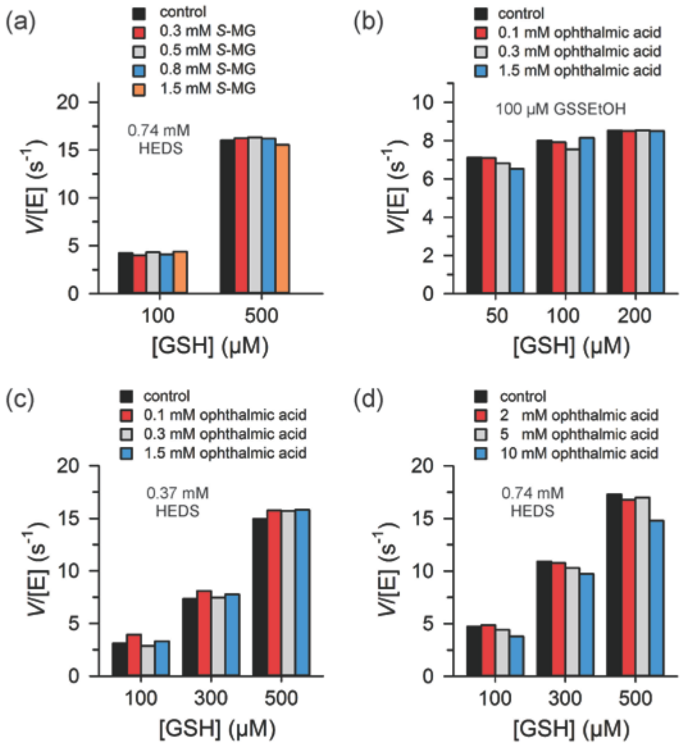

Supplementary Figure 10 | Competitive inhibition studies on ScGrx7 wild type enzyme. (**a**) Effect of up to 1.5 mM *S*-methylglutathione (*S*-MG) on the activity of ScGrx7 in the HEDS assay. Assays contained 0.74 mM HEDS and 0.1 or 0.5 mM GSH. (**b**) Effect of up to 1.5 mM ophthalmic acid on the activity of ScGrx7 in the GSSEtOH assay. Assays contained 0.1 mM GSSEtOH at three different GSH concentrations. (**c**) Effect of up to 1.5 mM ophthalmic acid on the activity of ScGrx7 in the HEDS assay. Assays contained 0.37 mM HEDS at three different GSH concentrations. (**d**) Effect of up to 10 mM ophthalmic acid on the activity of ScGrx7 in the HEDS assay. Assays contained 0.74 mM HEDS at three different GSH concentrations. All assays were started by the addition of enzyme.

Supplementary Figure 11

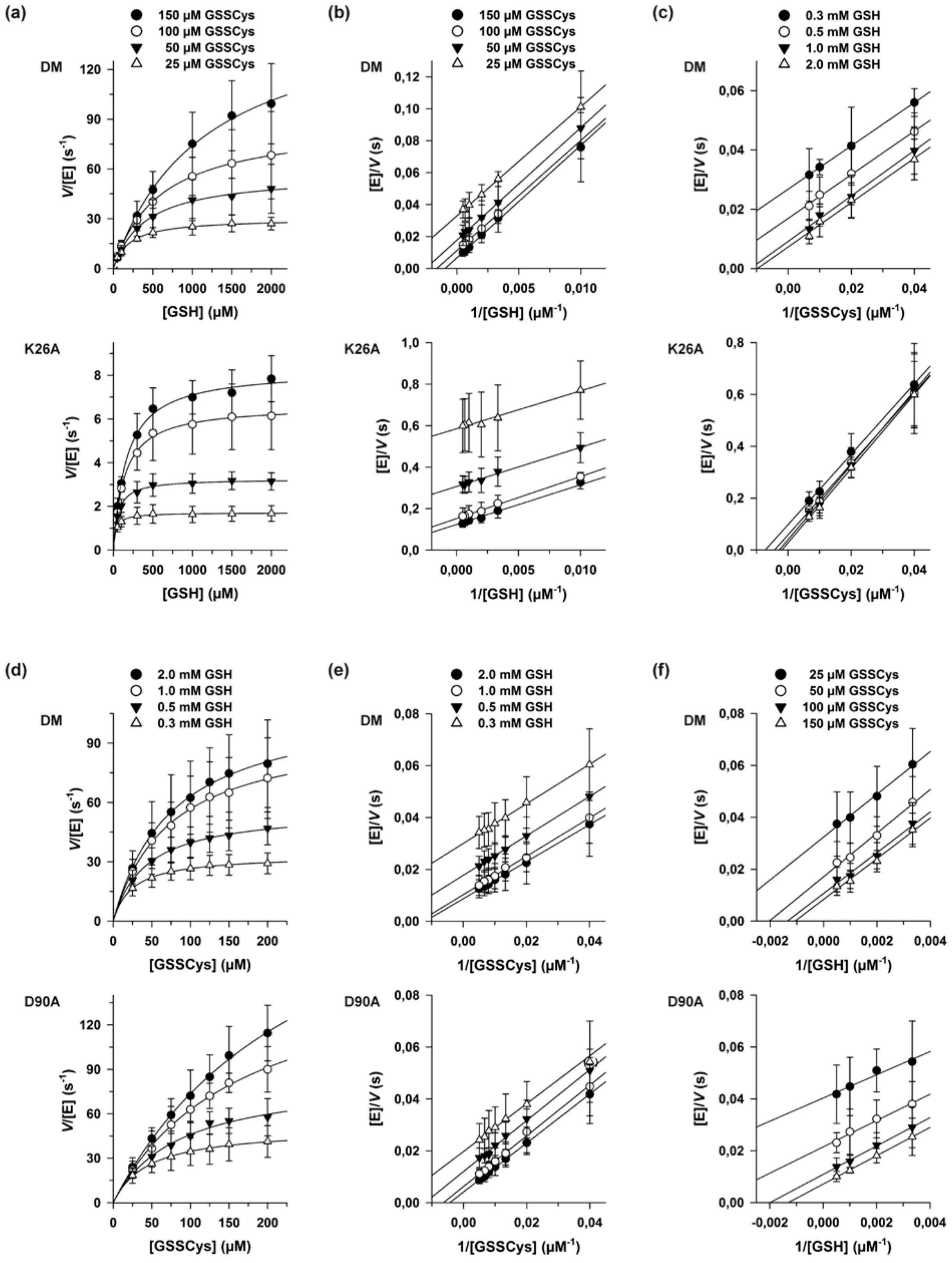

Supplementary Figure 11 | GSSCys assay steady-state kinetics of PfGrx$^{C32S/C88S}$ (DM) and the mutants K26A and D90A. (**a**) Michaelis-Menten plots of the GSH-dependent reaction velocity at different initial concentrations of GSSCys. (**b**) Lineweaver-Burk plots of the GSH-dependent reaction velocity at different initial concentrations of GSSCys revealing ping-pong kinetic patterns. (**c**) Lineweaver-Burk plots of the GSSCys-dependent reaction velocity at four selected initial concentrations of GSH. (**d**) Michaelis-Menten plots of the GSSCys-dependent reaction velocity at different initial concentrations of GSH. (**e**) Lineweaver-Burk plots of the GSSCys-dependent reaction velocity at different initial concentrations of GSH revealing ping-pong kinetic patterns. (**f**) Lineweaver-Burk plots of the GSH-dependent reaction velocity at four selected initial concentrations of GSSCys. All data points are the mean ± S.D. from at least three independent replicates and were averaged from at least three independent protein purification experiments and analyzed as described for ScGrx7. Selected apparent kinetic constants from non-linear regression analyses of Michaelis-Menten plots are listed in Supplementary Table 6. Estimated true kinetic constants are listed in Supplementary Table 8.

Supplementary Figure 12

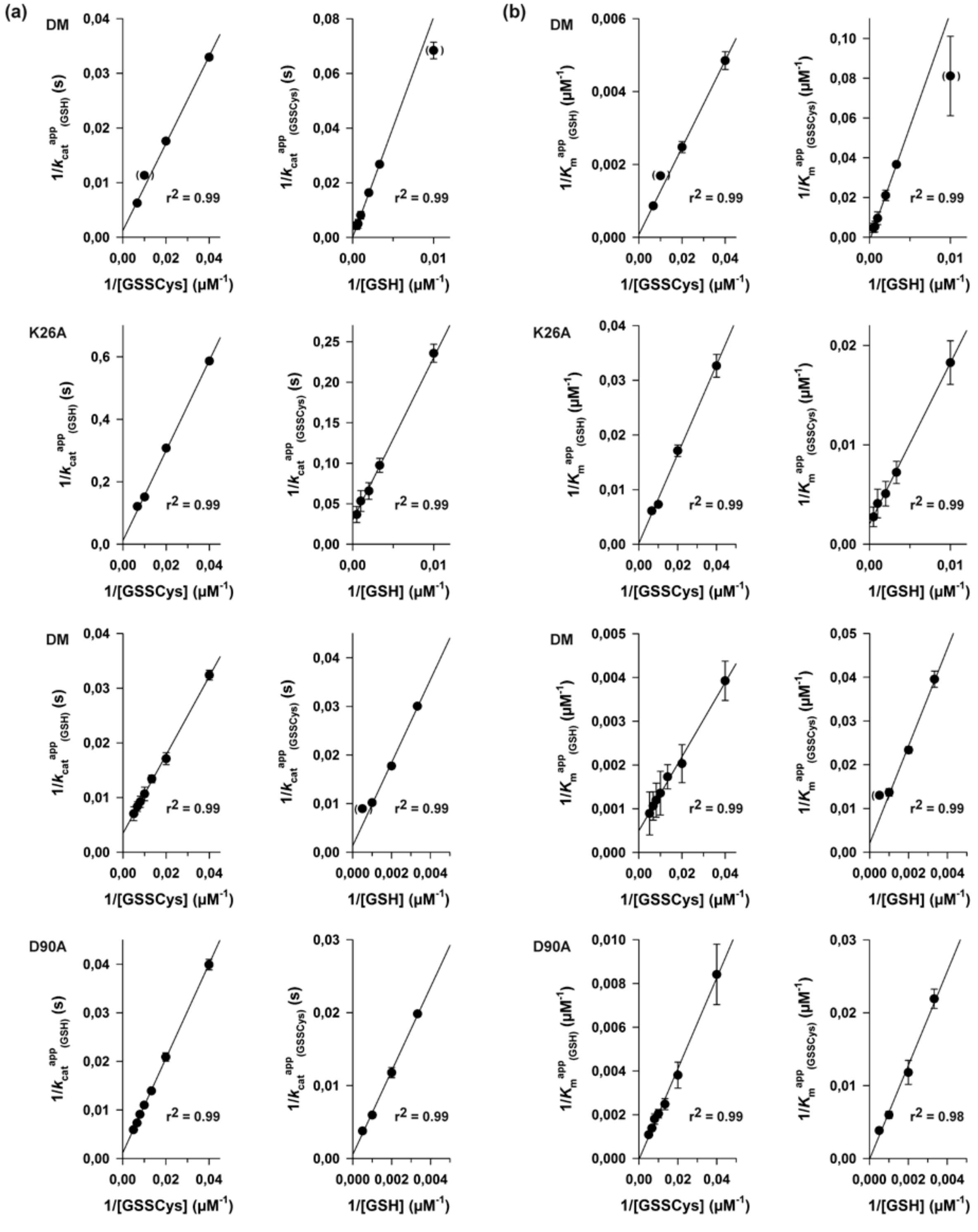

Supplementary Figure 12 | GSSCys assay secondary plots for PfGrx$^{C32S/C88S}$ (DM) and the mutants K26A and D90A. K_m^{app} and k_{cat}^{app} values were obtained from non-linear regression analyses of Michaelis-Menten plots (Supplementary Fig. 11). (**a**) Secondary plots of the k_{cat}^{app} values at different concentrations of GSSCys (left panels) and GSH (right panels) allowing the estimation of the true k_{cat} value from the y-axis intercept ($1/k_{cat}$) and of the true K_m value from the x-axis intercept ($-1/K_m$). (**b**) Secondary plots of the K_m^{app} values at different concentrations of GSSCys (left panels) and GSH (right panels). Outliers in brackets were identified based on the r^2 values and omitted from the linear regression analysis in Sigmaplot 12.5. Estimated true kinetic constants and Dalziel coefficients are listed in Supplementary Table 8.

Supplementary Figure 13

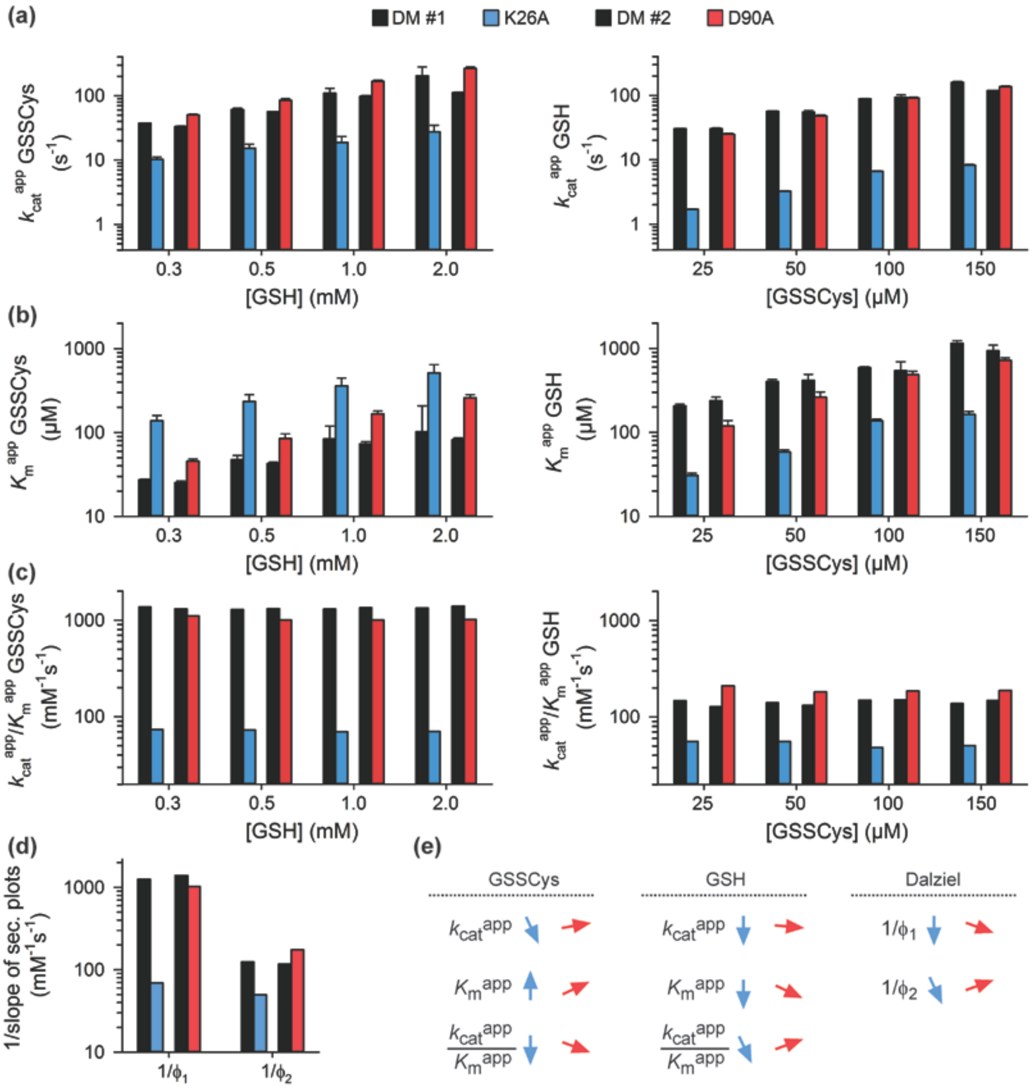

Supplementary Figure 13 | Conserved relevance of Lys26 and Asp90 of PfGrx for the GSSCys assay. (**a**) and (**b**) Selected k_{cat}^{app} and K_m^{app} values of $PfGrx^{C32S/C88S}$ (DM) and the mutants K26A and D90A for GSSCys and GSH. (**c**) Calculated catalytic efficiencies from panels **a** and **b**. (**d**) Reciprocal Dalziel coefficients obtained from Supplementary Fig. 12. (**e**) Summary of the altered kinetic parameters. The effects on the kinetic parameters are highly similar to the effects of the corresponding mutants of ScGrx7 summarized in Figs. 2 and 4. Original plots and kinetic parameters for panels **a**-**c** are shown in Supplementary Fig. 11 and Supplementary Table 6 and are the mean ± S.D. from at least three independent replicates. Statistical analyses and P-values for the k_{cat}^{app} and K_m^{app} values from panels **a** and **b** are listed in Supplementary Table 11. Reciprocal Dalziel coefficients for panel **d** and true k_{cat} values are listed in Supplementary Table 8.

Supplementary Figure 14

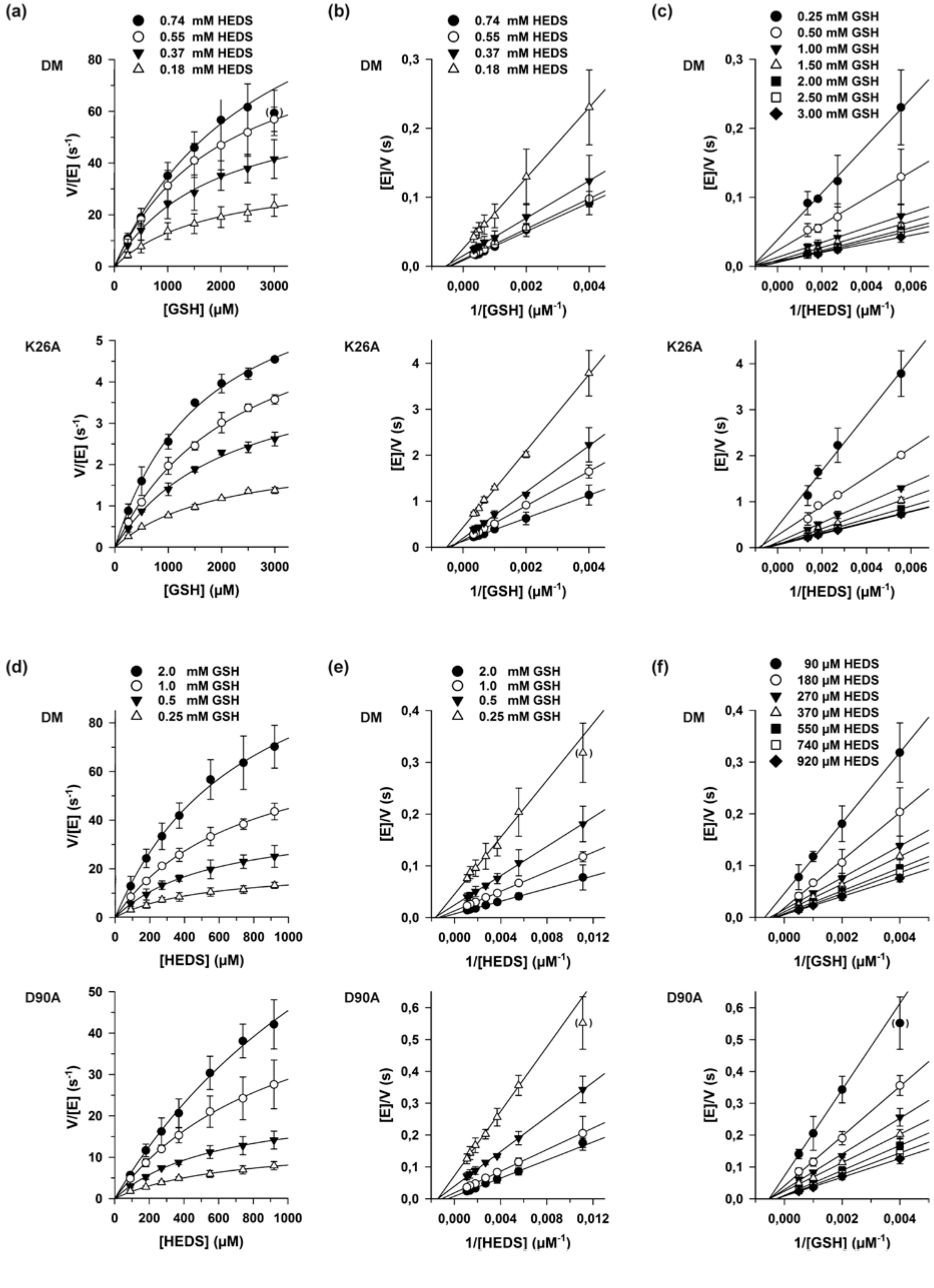

Supplementary Figure 14 | HEDS assay steady-state kinetics of PfGrx$^{C32S/C88S}$ (DM) and the mutants K26A and D90A. (**a**) Michaelis-Menten plots of the GSH-dependent reaction velocity at different initial concentrations of HEDS. (**b**) Lineweaver-Burk plots of the GSH-dependent reaction velocity at different initial concentrations of HEDS revealing sequential kinetic patterns. (**c**) Lineweaver-Burk plots of the HEDS-dependent reaction velocity at seven different initial concentrations of GSH. (**d**) Michaelis-Menten plots of the HEDS-dependent reaction velocity at different initial concentrations of GSH. (**e**) Lineweaver-Burk plots of the HEDS-dependent reaction velocity at different initial concentrations of GSH revealing sequential kinetic patterns. (**f**) Lineweaver-Burk plots of the GSH-dependent reaction velocity at seven different initial concentrations of HEDS. All data points are the mean ± S.D. from at least three independent replicates and were averaged from at least three independent protein purification experiments and analyzed as described for ScGrx7. Selected apparent kinetic constants from non-linear regression analyses of Michaelis-Menten plots are listed in Supplementary Table 7. Estimated true kinetic constants are listed in Supplementary Table 8.

Supplementary Figure 15

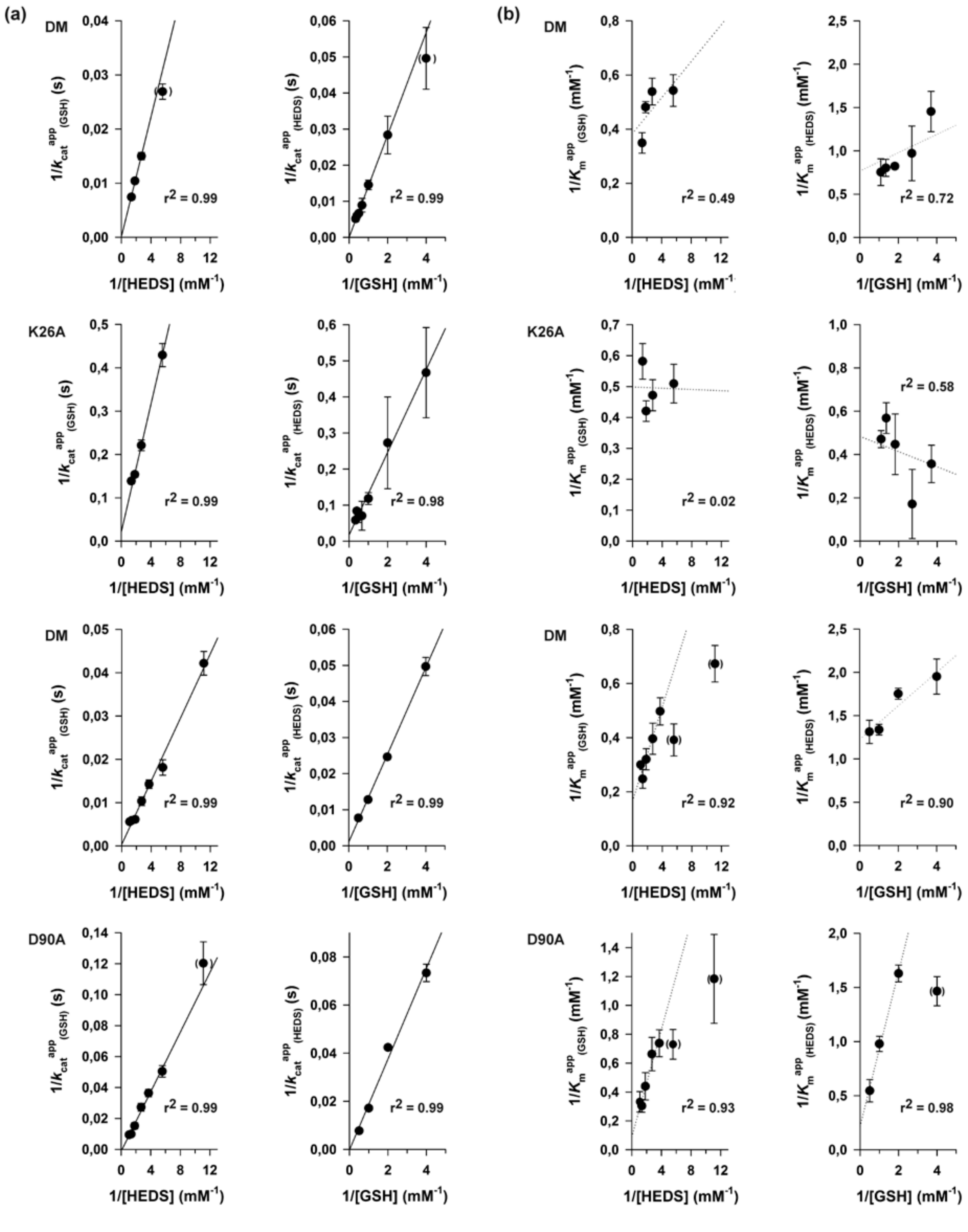

Supplementary Figure 15 | HEDS assay secondary plots for PfGrx$^{C32S/C88S}$ (DM) and the mutants K26A and D90A. K_m^{app} and k_{cat}^{app} values were obtained from non-linear regression analyses of Michaelis-Menten plots (Supplementary Fig. 14). (**a**) Secondary plots of the k_{cat}^{app} values at different concentrations of HEDS (left panels) and GSH (right panels) allowing the estimation of the true k_{cat} value from the y-axis intercept ($1/k_{cat}$) and of the true K_m value from the x-axis intercept ($-1/K_m$). Outliers in brackets at the lowest substrate concentration were identified based on the r^2 values and omitted from the linear regression analysis in Sigmaplot 12.5. (**b**) Secondary plots of the K_m^{app} values at different concentrations of HEDS (left panels) and GSH (right panels). Please note that most of the differences for K_m^{app} values among independent measurements and mutants are not statistically significant (see also Supplementary Table 11). Estimated true kinetic constants and Dalziel coefficients are listed in Supplementary Table 8.

Supplementary Figure 16

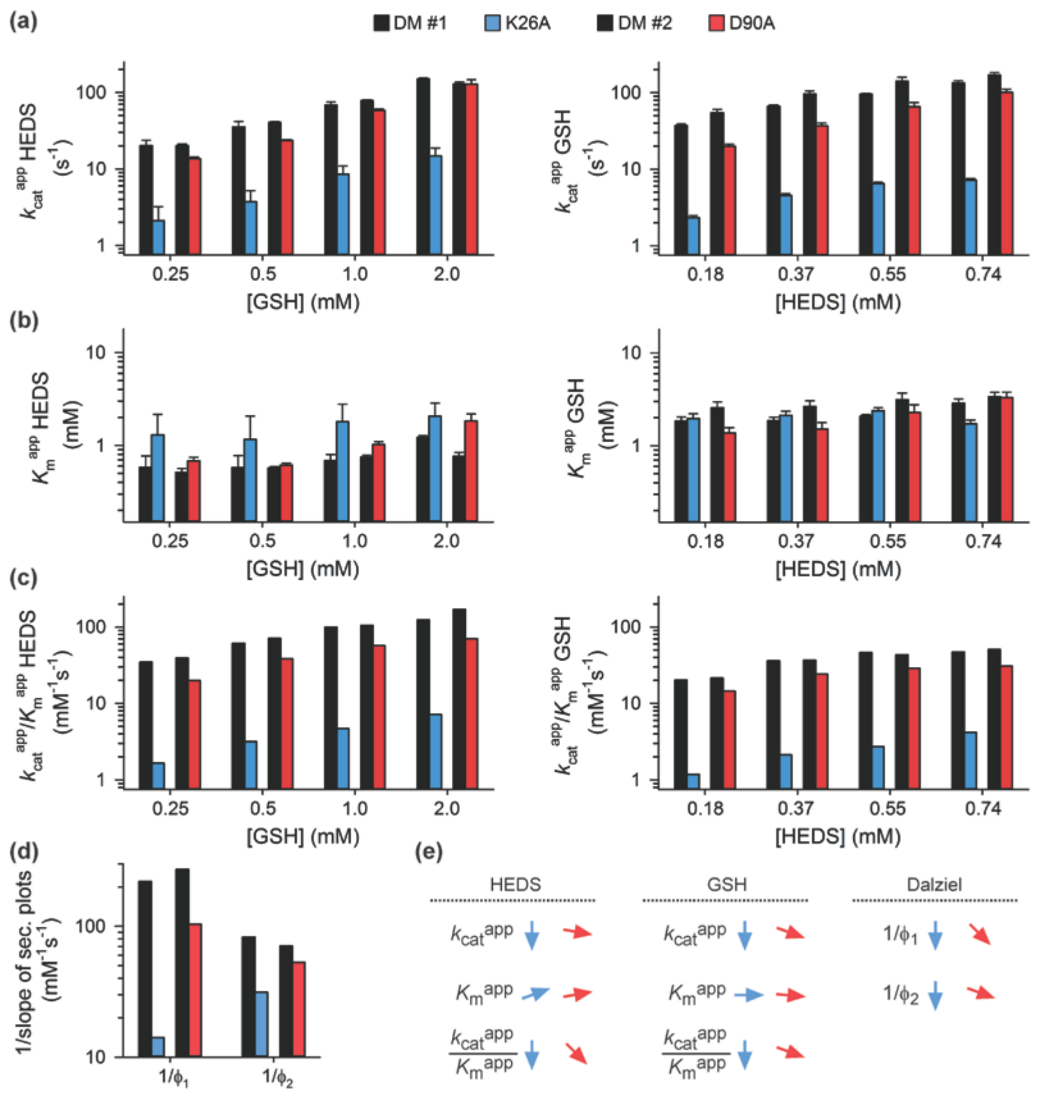

Supplementary Figure 16 | Conserved relevance of Lys26 and Asp90 of PfGrx for the HEDS assay. (**a**) and (**b**) Selected k_{cat}^{app} and K_m^{app} values of PfGrx$^{C32S/C88S}$ (DM) and the mutants K26A and D90A for HEDS and GSH. (**c**) Calculated catalytic efficiencies from panels **a** and **b**. (**d**) Reciprocal Dalziel coefficients obtained from Supplementary Fig. 15. (**e**) Summary of the altered kinetic parameters. The effects on the kinetic parameters are highly similar to the effects of the corresponding mutants of ScGrx7 summarized in Figs. 3 and 5. Original plots and kinetic parameters for panels **a**-**c** are shown in Supplementary Fig. 14 and Supplementary Table 7 and are the mean ± S.D. from at least three independent replicates. Statistical analyses and P-values for the k_{cat}^{app} and K_m^{app} values from panels **a** and **b** are listed in Supplementary Table 11. Reciprocal Dalziel coefficients for panel **d** and true k_{cat} values are listed in Supplementary Table 8.

Supplementary Figure 17

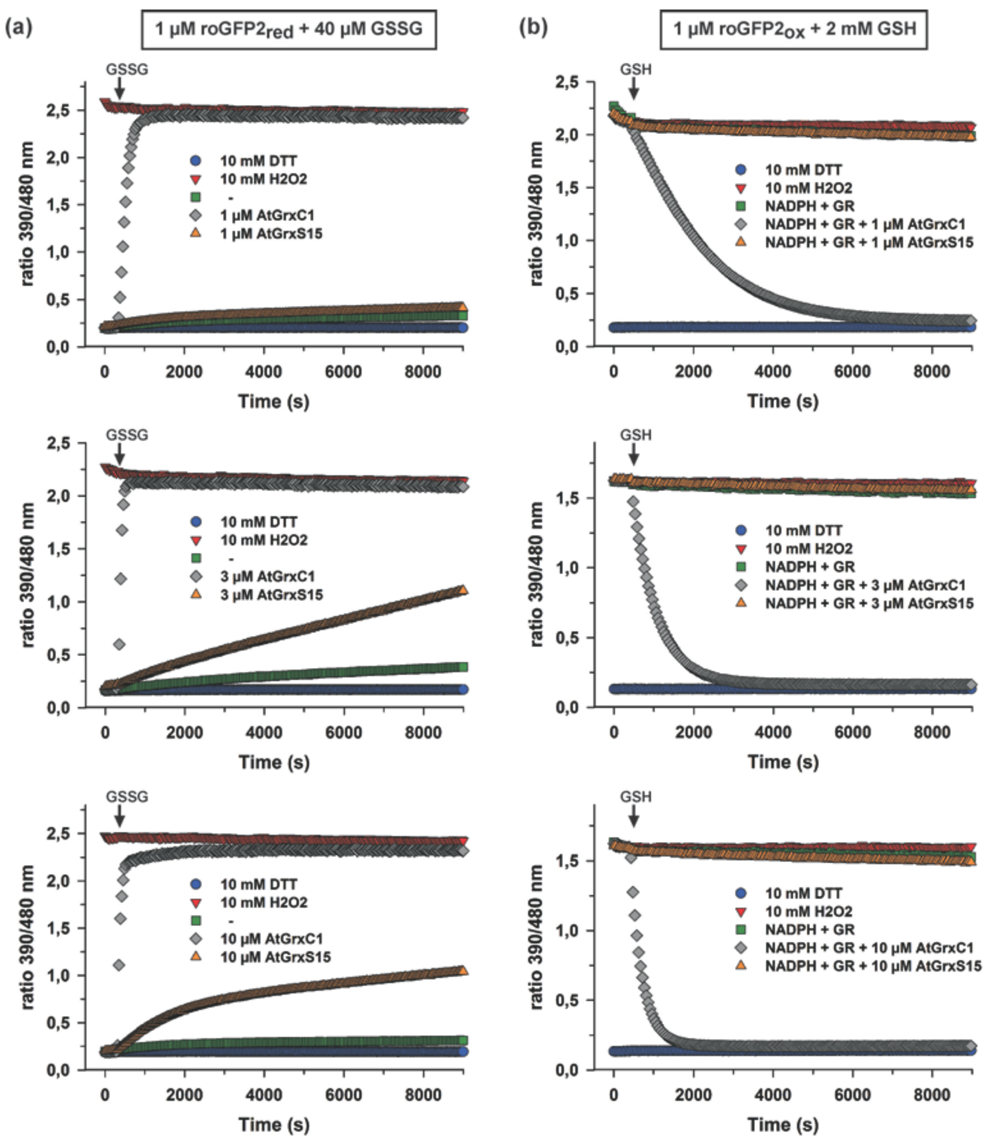

Supplementary Figure 17 | Grx-catalyzed redox reactions of roGFP2. (**a**) GSSG-dependent oxidation kinetics of 1 µM reduced roGFP2 in the presence of three different concentrations of AtGrxS15. (**b**) GSH-dependent reduction kinetics of 1 µM oxidized roGFP2 in the presence of three different concentrations of AtGrxS15. Samples containing the enzymatically active dithiol glutaredoxin AtGrxC1 served as positive controls. Negative controls contained roGFP2 without glutaredoxin. Samples with DTT or H_2O_2 were used to fully oxidize or reduce roGFP2. Gain settings for the detection of the roGFP2 fluorescence after excitation at 390 and 480 nm were always adjusted before each experiment resulting in different absolute ratio values calculated for each experiment.

Supplementary Figure 18

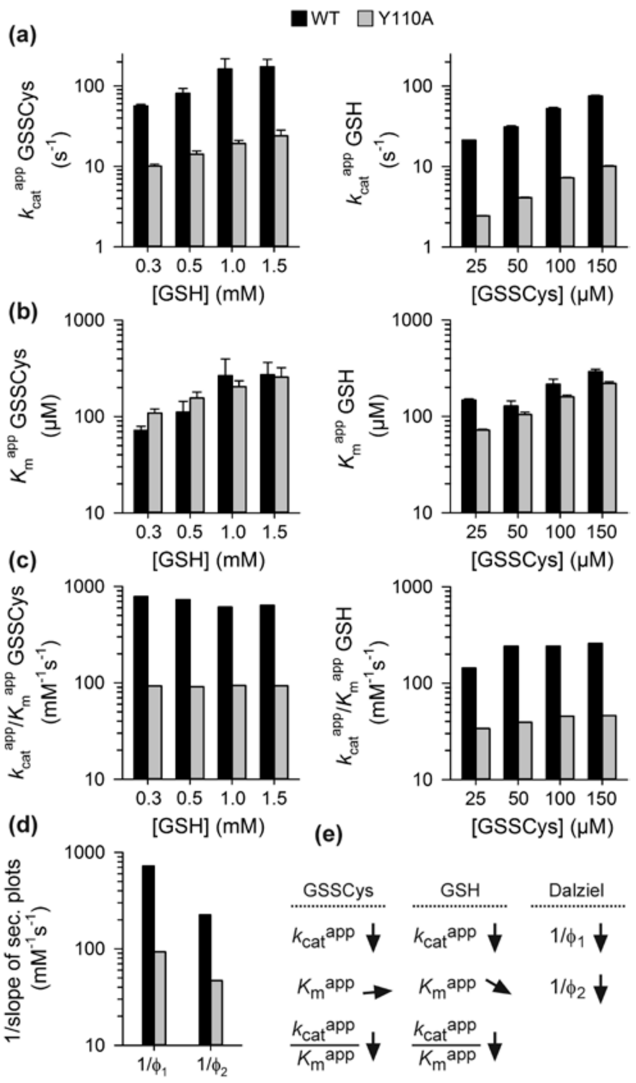

Supplementary Figure 18 | Relevance of Tyr110 of ScGrx7 for the steady-state kinetics with GSSCys. (**a**) and (**b**) Preliminary k_{cat}^{app} and K_m^{app} values of Y110A for GSSCys and GSH. (**c**) Calculated catalytic efficiencies from panels **a** and **b**. (**d**) Reciprocal Dalziel coefficients, which probably reflect the rate constants of the oxidative and reductive half-reaction with GSSCys and GSH, respectively. (**e**) Summary of the altered kinetic parameters for Y110A as compared to wild type enzyme. Data in panels **a** and **b** are the mean ± S.D. from at least three independent replicates and were obtained from three independent protein purifications.

Supplementary Figure 19

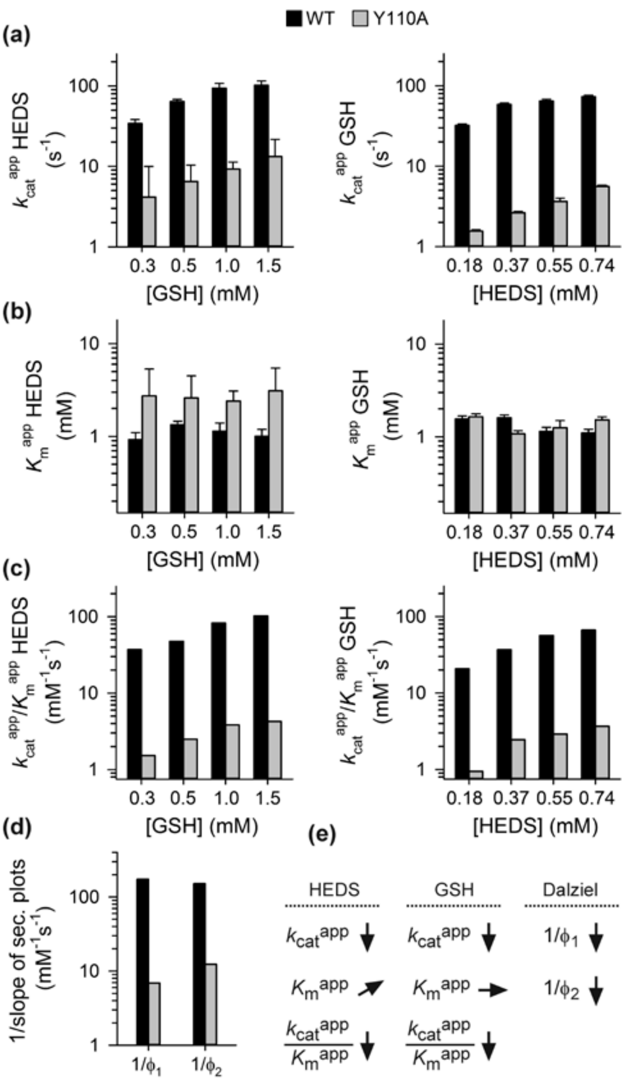

Supplementary Figure 19 | Relevance of Tyr110 of ScGrx7 for the steady-state kinetics with HEDS. (**a**) and (**b**) Preliminary k_{cat}^{app} and K_m^{app} values of Y110A for HEDS and GSH. (**c**) Calculated catalytic efficiencies from panels **a** and **b**. (**d**) Reciprocal Dalziel coefficients. (**e**) Summary of the altered kinetic parameters for Y110A as compared to wild type enzyme. Data in panels **a** and **b** are the mean ± S.D. from at least three independent replicates and were obtained from three independent protein purifications.

Supplementary Table 1. Comparison of estimated true k_{cat} and K_m values and Dalziel coefficients Φ_1 and Φ_2 of wild type and mutant ScGrx7 in the GSSCys and HEDS assay.

GSSCys assay

**ScGrx7**	**1/Φ_1** [a] ($M^{-1}s^{-1}$)	**1/Φ_2** [a] ($M^{-1}s^{-1}$)	**k_{cat}** [a] (s^{-1})	**$K_{m\ (GSH)}$** [a] (mM)	**$K_{m\ (GSSCys)}$** [a] (mM)
WT	7.3×10^5	4.7×10^5	∞	∞	∞
K105R	7.9×10^4	5.5×10^5	∞	∞	∞
K105A	3.6×10^4	1.5×10^5	∞	∞	∞
K105Y	2.1×10^4	1.2×10^5	∞	∞	∞
K105E	6.1×10^3	2.8×10^4	∞	∞	∞
WT	9.7×10^5	2.5×10^5	∞	∞	∞
E170D	9.3×10^5	1.9×10^5	∞	∞	∞
E170A	5.3×10^5	2.8×10^5	∞	∞	∞
E170K	5.4×10^5	2.9×10^5	∞	∞	∞

HEDS assay

**ScGrx7**	**1/Φ_1** [b] ($M^{-1}s^{-1}$)	**1/Φ_2** [b] ($M^{-1}s^{-1}$)	**k_{cat}** [b] (s^{-1})	**$K_{m\ (GSH)}$** [c] (mM)	**$K_{m\ (HEDS)}$** [c] (mM)
WT	1.6×10^5	2.4×10^5	273	1.2 ± 0.1	2.3 ± 0.7
K105R	1.8×10^4	4.9×10^4	26	1.5 ± 0.2	2.7 ± 0.4
K105A	7.6×10^3	2.3×10^4	13	1.3 ± 0.1	2.5 ± 0.4
K105Y	6.2×10^3	1.0×10^4	8	1.4 ± 0.1	2.2 ± 0.2
K105E	1.2×10^3	1.6×10^3	2	1.4 ± 0.1	2.5 ± 0.6
WT	2.2×10^5	1.2×10^5	1367 - ∞	2.3 ± 0.5	1.5 ± 0.2
E170D	1.6×10^5	1.2×10^5	1263 - ∞	1.8 ± 0.4	1.5 ± 0.3
E170A	9.6×10^4	1.2×10^5	429	2.0 ± 0.1	2.7 ± 0.2
E170K	1.1×10^5	1.2×10^5	428	2.1 ± 0.2	2.5 ± 0.3

[a] Estimated k_{cat}, K_m values and Dalziel coefficients were obtained from Supplementary Figs. 3 and 7.
[b] Estimated k_{cat} values and Dalziel coefficients were obtained from Supplementary Figs. 5a and 9a.
[c] Estimated K_m values were averaged from Supplementary Figs. 4 and 8.

Supplementary Table 2. Selected k_{cat}^{app} and K_m^{app} values from GSSCys assays with ScGrx7 wild type enzyme and Lys105 mutants obtained from Supplementary Fig. 2.

ScGrx7	[GSSCys] (µM)	$k_{cat}^{app}{}_{(GSH)}$ [a] (s^{-1})		$K_m^{app}{}_{(GSH)}$ [a] (µM)		$k_{cat}^{app}/K_m^{app}{}_{(GSH)}$ (mM^{-1}s^{-1})	
WT	25	18.7 ± 0.1	100 %	43.3 ± 1.3	100 %	432	100 %
K105R	25	2.0 ± 0.01	11 %	4.1 ± 0.6	9 %	485	112 %
K105A	25	0.9 ± 0.01	5 %	5.4 ± 1.3	12 %	169	39 %
K105Y	25	0.5 ± 0.01	3 %	4.1 ± 1.2	9 %	126	29 %
K105E	25	0.2 ± 0.00	1 %	6.4 ± 1.0	15 %	25	6 %
WT	50	40.4 ± 0.3	100 %	100 ± 3.0	100 %	403	100 %
K105R	50	4.0 ± 0.02	10 %	11.2 ± 0.8	11 %	360	89 %
K105A	50	1.9 ± 0.02	5 %	14.5 ± 1.4	15 %	128	32 %
K105Y	50	1.0 ± 0.01	3 %	5.5 ± 0.5	6 %	181	45 %
K105E	50	0.3 ± 0.01	1 %	9.9 ± 0.5	10 %	30	8 %
WT	100	80.1 ± 1.6	100 %	182 ± 12	100 %	440	100 %
K105R	100	7.9 ± 0.1	10 %	14.8 ± 1.6	8 %	534	121 %
K105A	100	3.6 ± 0.03	5 %	27.0 ± 1.6	15 %	133	30 %
K105Y	100	2.0 ± 0.02	3 %	11.6 ± 1.9	6 %	170	39 %
K105E	100	0.6 ± 0.01	1 %	17.6 ± 1.6	10 %	32	7 %
WT	150	127 ± 1.9	100 %	281 ± 12	100 %	452	100 %
K105R	150	11.6 ± 0.1	9 %	22.7 ± 1.1	8 %	513	113 %
K105A	150	5.3 ± 0.1	4 %	33.9 ± 2.7	12 %	158	35 %
K105Y	150	2.9 ± 0.1	2 %	19.7 ± 2.6	7 %	145	32 %
K105E	150	0.9 ± 0.01	1 %	31.7 ± 2.7	11 %	28	6 %

ScGrx7	[GSH] (µM)	$k_{cat}^{app}{}_{(GSSCys)}$ [a] (s^{-1})		$K_m^{app}{}_{(GSSCys)}$ [a] (µM)		$k_{cat}^{app}/K_m^{app}{}_{(GSSCys)}$ (mM^{-1}s^{-1})	
WT	50	23.9 ± 0.9	100 %	36.2 ± 4.0	100 %	661	100 %
K105R	50	24.7 ± 4.0	103 %	311 ± 69	858 %	80	12 %
K105A	50	8.4 ± 1.4	35 %	247 ± 61	683 %	34	5 %
K105Y	50	5.2 ± 0.7	22 %	237 ± 47	654 %	22	3 %
K105E	50	1.2 ± 0.05	5 %	188 ± 12	518 %	7	1 %
WT	100	49.0 ± 0.3	100 %	69.2 ± 1.0	100 %	709	100 %
K105R	100	51.1 ± 2.3	104 %	657 ± 35	949 %	78	11 %
K105A	100	14.3 ± 2.0	29 %	401 ± 71	580 %	36	5 %
K105Y	100	11.5 ± 0.4	23 %	557 ± 22	806 %	21	3 %
K105E	100	2.2 ± 0.2	5 %	358 ± 46	517 %	6	1 %
WT	200	111 ± 5.1	100 %	160 ± 9.0	100 %	692	100 %
K105R	200	66.9 ± 14	60 %	810 ± 189	508 %	83	12 %
K105A	200	26.9 ± 1.3	24 %	736 ± 42	461 %	37	5 %
K105Y	200	23.9 ± 3.0	22 %	1194 ± 167	748 %	20	3 %
K105E	200	3.1 ± 0.3	3 %	492 ± 67	308 %	6	1 %
WT	300	144 ± 5.1	100 %	192 ± 10.8	100 %	749	100 %
K105R	300	152 ± 44	106 %	1934 ± 595	1008 %	79	12 %
K105A	300	48.7 ± 16	34 %	1346 ± 488	702 %	36	5 %
K105Y	300	17.5 ± 5.2	12 %	810 ± 276	422 %	22	3 %
K105E	300	13.4 ± 0.4	2 %	527 ± 77	275 %	7	1 %

[a] Mean±SD from Michaelis-Menten plots of at least two independent protein purifications. Percentages are relative to wild type enzyme. P-values are listed in Supplementary Table 11.

Supplementary Table 3. Selected k_{cat}^{app} and K_m^{app} values from HEDS assays with ScGrx7 wild type enzyme and Lys105 mutants obtained from Supplementary Fig. 4.

**ScGrx7**	**[HEDS]** (mM)	$k_{cat}^{app}{}_{(GSH)}$ [a] (s^{-1})		$K_m^{app}{}_{(GSH)}$ [a] (mM)		$k_{cat}^{app}/K_m^{app}{}_{(GSH)}$ ($mM^{-1}s^{-1}$)	
WT	0.18	26.1 ± 2.1	100 %	1.21 ± 0.20	100 %	21.5	100 %
K105R	0.18	2.9 ± 0.07	11 %	1.62 ± 0.07	134 %	1.8	8 %
K105A	0.18	1.0 ± 0.02	4 %	1.22 ± 0.04	101 %	0.8	4 %
K105Y	0.18	1.0 ± 0.04	4 %	1.45 ± 0.12	119 %	0.7	3 %
K105E	0.18	0.2 ± 0.01	1 %	1.53 ± 0.13	126 %	0.1	1 %
WT	0.37	50.1 ± 3.2	100 %	1.23 ± 0.15	100 %	40.6	100 %
K105R	0.37	5.6 ± 0.09	11 %	1.64 ± 0.05	133 %	3.4	8 %
K105A	0.37	2.3 ± 0.03	5 %	1.39 ± 0.04	113 %	1.6	4 %
K105Y	0.37	1.8 ± 0.03	4 %	1.48 ± 0.04	120 %	1.2	3 %
K105E	0.37	0.4 ± 0.01	1 %	1.38 ± 0.07	112 %	0.3	1 %
WT	0.55	66.4 ± 3.2	100 %	1.12 ± 0.11	100 %	59.3	100 %
K105R	0.55	7.6 ± 0.22	11 %	1.48 ± 0.08	132 %	5.2	9 %
K105A	0.55	3.2 ± 0.12	5 %	1.24 ± 0.09	111 %	2.6	4 %
K105Y	0.55	2.4 ± 0.07	4 %	1.36 ± 0.08	122 %	1.7	3 %
K105E	0.55	0.5 ± 0.03	1 %	1.46 ± 0.17	130 %	0.3	1 %
WT	0.74	81.5 ± 4.7	100 %	1.12 ± 0.13	100 %	73.1	100 %
K105R	0.74	8.4 ± 0.6	10 %	1.22 ± 0.18	110 %	6.9	9 %
K105A	0.74	3.9 ± 0.1	5 %	1.33 ± 0.08	119 %	2.9	4 %
K105Y	0.74	2.9 ± 0.1	4 %	1.21 ± 0.06	108 %	2.4	3 %
K105E	0.74	0.6 ± 0.00	1 %	1.29 ± 0.15	116 %	0.5	1 %

**ScGrx7**	**[GSH]** (mM)	$k_{cat}^{app}{}_{(HEDS)}$ [a] (s^{-1})		$K_m^{app}{}_{(HEDS)}$ [a] (mM)		$k_{cat}^{app}/K_m^{app}{}_{(HEDS)}$ ($mM^{-1}s^{-1}$)	
WT	0.3	66.8 ± 34	100 %	2.16 ± 1 4	100 %	30.9	100 %
K105R	0.3	10.1 ± 1.8	15 %	2.86 ± 0.8	132 %	3.5	11 %
K105A	0.3	2.7 ± 0.5	4 %	2.14 ± 0.5	99 %	1.3	4 %
K105Y	0.3	2.2 ± 0.5	3 %	2.28 ± 0.6	106 %	1.0	3 %
K105E	0.3	1.0 ± 0.7	2 %	2.31 ± 1.3	94 %	0.4	1 %
WT	0.5	91.3 ± 7.9	100 %	2.05 ± 0.2	100 %	44.5	100 %
K105R	0.5	14.0 ± 6.2	15 %	3.37 ± 1.8	164 %	4.2	9 %
K105A	0.5	4.6 ± 1.8	5 %	2.53 ± 1.2	123 %	1.8	4 %
K105Y	0.5	3.1 ± 0.4	3 %	2.18 ± 0.3	106 %	1.4	3 %
K105E	0.5	1.3 ± 1.0	1 %	2.14 ± 4.3	96 %	0.6	1 %
WT	1.0	137 ± 15	100 %	1.82 ± 0.3	100 %	75.2	100 %
K105R	1.0	18.6 ± 3.8	14 %	2.82 ± 0.7	154 %	6.6	9 %
K105A	1.0	8.2 ± 1.3	6 %	2.89 ± 0.6	158 %	2.8	4 %
K105Y	1.0	5.2 ± 1.2	4 %	2.22 ± 0.7	122 %	2.3	3 %
K105E	1.0	1.4 ± 0.2	1 %	3.07 ± 0.6	168 %	0.4	1 %
WT	1.5	310 ± 77	100 %	3.72 ± 1.1	100 %	83.3	100 %
K105R	1.5	23.4 ± 3.4	8 %	2.79 ± 0.5	75 %	8.4	10 %
K105A	1.5	13.5 ± 4.4	8 %	3.83 ± 1.5	103 %	3.5	4 %
K105Y	1.5	5.3 ± 0.1	2 %	1.76 ± 0.5	47 %	3.0	4 %
K105E	1.5	2.4 ± 1.0	1 %	4.25 ± 1.3	114 %	0.6	1 %

[a] Mean±SD from Michaelis-Menten plots of at least three independent protein purifications. Percentages are relative to wild type enzyme. P-values are listed in Supplementary Table 11.

Supplementary Table 4. Selected k_{cat}^{app} and K_m^{app} values from GSSCys assays with ScGrx7 wild type enzyme and Glu170 mutants obtained from Supplementary Fig. 6.

ScGrx7	[GSSCys] (µM)	$k_{cat}^{app}{}_{(GSH)}$ [a] (s^{-1})		$K_m^{app}{}_{(GSH)}$ [a] (µM)		$k_{cat}^{app}/K_m^{app}{}_{(GSH)}$ (mM^{-1}s^{-1})	
WT	25	24.3 ± 0.5	100 %	120 ± 8.4	100 %	202	100 %
E170D	25	23.3 ± 0.8	96 %	160 ± 17	133 %	146	72 %
E170A	25	14.7 ± 0.3	61 %	71.1 ± 5.9	59 %	207	103 %
E170K	25	13.2 ± 0.2	54 %	50.7 ± 4.5	42 %	261	129 %
WT	50	48.6 ± 1.1	100 %	223 ± 14	100 %	218	100 %
E170D	50	46.7 ± 1.7	96 %	277 ± 25	124 %	169	77 %
E170A	50	27.2 ± 1.3	56 %	106 ± 19	47 %	258	118 %
E170K	50	26.1 ± 0.6	54 %	92.4 ± 7.6	41 %	283	130 %
WT	100	99.8 ± 2.5	100 %	436 ± 24	100 %	229	100 %
E170D	100	96.0 ± 2.2	89 %	533 ± 25	110 %	180	81 %
E170A	100	55.0 ± 2.7	45 %	200 ± 27	29 %	275	159 %
E170K	100	51.6 ± 0.9	51 %	178 ± 9.0	40 %	290	128 %
WT	150	146 ± 7.5	100 %	638 ± 63	100 %	228	100 %
E170D	150	143 ± 4.6	96 %	771 ± 45	122 %	185	79 %
E170A	150	79.4 ± 2.7	55 %	278 ± 23	46 %	285	120 %
E170K	150	75.7 ± 0.5	52 %	257 ± 4.0	41%	295	127 %

ScGrx7	[GSH] (mM)	$k_{cat}^{app}{}_{(GSSCys)}$ [a] (s^{-1})		$K_m^{app}{}_{(GSSCys)}$ [a] (µM)		$k_{cat}^{app}/K_m^{app}{}_{(GSSCys)}$ (mM^{-1}s^{-1})	
WT	0.1	24.2 ± 0.4	100 %	30.2 ± 1.7	100 %	801	100 %
E170D	0.1	18.8 ± 0.3	78 %	28.2 ± 1.9	93 %	668	83 %
E170A	0.1	28.8 ± 0.5	119 %	62.7 ± 2.9	207 %	459	57 %
E170K	0.1	28.7 ± 0.4	119 %	57.3 ± 2.2	189 %	502	63 %
WT	0.2	50.2 ± 1.3	100 %	59.2 ± 4.2	100 %	847	100 %
E170D	0.2	39.9 ± 0.8	80 %	56.4 ± 3.3	95 %	707	84 %
E170A	0.2	54.4 ± 0.9	108 %	103 ± 3.5	173 %	520	63 %
E170K	0.2	58.2 ± 1.1	116 %	116 ± 4.5	196 %	503	59 %
WT	0.3	65.4 ± 1.1	100 %	67.5 ± 3.0	100 %	969	100 %
E170D	0.3	58.6 ± 1.5	90 %	68.2 ± 4.4	101 %	860	89 %
E170A	0.3	81.2 ± 2.9	124 %	140 ± 9.5	207 %	582	60 %
E170K	0.3	84.2 ± 1.5	129 %	156 ± 5.0	231 %	539	56 %
WT	1.0	225 ± 6.0	100 %	229 ± 9.6	100 %	985	100 %
E170D	1.0	204 ± 11	91 %	234 ± 20	102 %	871	88 %
E170A	1.0	236 ± 11	105 %	424 ± 27	186 %	557	57 %
E170K	1.0	242 ± 5.7	107 %	456 ± 14	199 %	531	54 %

[a] Mean±SD from Michaelis-Menten plots of at least two independent protein purifications. Percentages are relative to wild type enzyme. P-values are listed in Supplementary Table 11.

Supplementary Table 5. Selected k_{cat}^{app} and K_m^{app} values from HEDS assays with ScGrx7 wild type enzyme and Glu170 mutants obtained from Supplementary Fig. 8.

ScGrx7	[HEDS] (mM)	$k_{cat}^{app}{}_{(GSH)}$ [a] (s^{-1})		$K_m^{app}{}_{(GSH)}$ [a] (mM)		$k_{cat}^{app}/K_m^{app}{}_{(GSH)}$ ($mM^{-1}s^{-1}$)	
WT	0.18	39.0 ± 2.2	100 %	2.34 ± 0.2	100 %	16.7	100 %
E170D	0.18	30.1 ± 1.9	77 %	1.79 ± 0.2	76 %	16.8	101 %
E170A	0.18	16.3 ± 0.7	42 %	1.91 ± 0.1	85 %	8.3	50 %
E170K	0.18	19.3 ± 0.4	50 %	2.06 ± 0.1	88 %	9.4	56 %
WT	0.37	71.7 ± 3.7	100 %	2.68 ± 0.2	100 %	26.7	100 %
E170D	0.37	63.2 ± 2.5	88 %	2.13 ± 0.1	79 %	29.7	111 %
E170A	0.37	32.1 ± 1.4	45 %	2.05 ± 0.1	77 %	15.6	58 %
E170K	0.37	36.8 ± 0.4	51 %	2.08 ± 0.0	77 %	17.7	66 %
WT	0.55	115 ± 4.5	100 %	2.85 ± 0.2	100 %	40.3	100 %
E170D	0.55	90.6 ± 4.9	79 %	2.24 ± 0.2	79 %	40.4	100 %
E170A	0.55	47.9 ± 2.2	42 %	2.18 ± 0.2	77 %	22.0	55 %
E170K	0.55	53.0 ± 1.1	46 %	2.11 ± 0.1	74 %	25.1	62 %
WT	0.74	161 ± 9.5	100 %	3.36 ± 0.3	100 %	47.9	100 %
E170D	0.74	119 ± 11	74 %	2.46 ± 0.3	73 %	48.2	101 %
E170A	0.74	58.4 ± 1.1	36 %	2.08 ± 0.1	62 %	28.0	59 %
E170K	0.74	75.8 ± 0.7	47 %	2.49 ± 0.0	74 %	30.4	64 %

ScGrx7	[GSH] (mM)	$k_{cat}^{app}{}_{(HEDS)}$ [a] (s^{-1})		$K_m^{app}{}_{(HEDS)}$ [a] (mM)		$k_{cat}^{app}/K_m^{app}{}_{(HEDS)}$ ($mM^{-1}s^{-1}$)	
WT	0.3	37.8 ± 1.1	100 %	1.38 ± 0.1	100 %	27.3	100 %
E170D	0.3	30.2 ± 0.7	80 %	1.07 ± 0.1	77 %	28.3	103 %
E170A	0.3	31.9 ± 1.2	84 %	2.42 ± 0.1	175 %	13.2	48 %
E170K	0.3	34.5 ± 1.2	91 %	2.37 ± 0.1	171 %	14.6	53 %
WT	0.5	57.0 ± 0.9	100 %	1.26 ± 0.1	100 %	45.2	100 %
E170D	0.5	55.8 ± 1.5	98 %	1.32 ± 0.1	105 %	42.2	93 %
E170A	0.5	54.7 ± 1.6	96 %	2.88 ± 0.1	229 %	19.0	42 %
E170K	0.5	53.8 ± 1.3	94 %	2.39 ± 0.1	189 %	22.5	50 %
WT	1.0	115 ± 3.4	100 %	1.59 ± 0.1	100 %	72.7	100 %
E170D	1.0	113 ± 3.0	98 %	1.65 ± 0.1	104 %	68.1	94 %
E170A	1.0	91.5 ± 2.7	79 %	2.82 ± 0.1	178 %	32.4	45 %
E170K	1.0	92.4 ± 3.4	80 %	2.45 ± 0.1	154 %	37.9	52 %
WT	1.5	163 ± 8.8	100 %	1.73 ± 0.1	100 %	94.4	100 %
E170D	1.5	154 ± 3.7	94 %	1.81 ± 0.1	105 %	84.8	90 %
E170A	1.5	117 ± 6.2	71 %	2.78 ± 0.2	161 %	42.0	44 %
E170K	1.5	139 ± 8.2	85 %	2.90 ± 0.2	168 %	47.8	51 %

[a] Mean±SD from Michaelis-Menten plots of at least three independent protein purifications. Percentages are relative to wild type enzyme. P-values are listed in Supplementary Table 11.

Supplementary Table 6. Selected k_{cat}^{app} and K_m^{app} values from GSSCys assays with PfGrx$^{C32S/C88S}$ and the mutants K26A and D90A obtained from Supplementary Fig. 11.

PfGrx	[GSSCys] (µM)	$k_{cat}^{app}{}_{(GSH)}$ [a] (s^{-1})		$K_m^{app}{}_{(GSH)}$ [a] (µM)		$k_{cat}^{app}/K_m^{app}{}_{(GSH)}$ (mM^{-1}s^{-1})	
DM	25	30.4 ± 0.4	100 %	206 ± 10	100 %	147	100 %
K26A	25	1.7 ± 0.1	6 %	31.2 ± 2.0	15 %	55	37 %
D90A	25	25.1 ± 0.7	83 %	119 ± 19	58 %	211	144 %
DM	50	56.8 ± 1.1	100 %	403 ± 25	100 %	141	100 %
K26A	50	3.3 ± 0.1	6 %	61.6 ± 3.6	15 %	53	38 %
D90A	50	47.8 ± 2.0	84 %	262 ± 41	65 %	182	129 %
DM	100	88.3 ± 0.9	100 %	591 ± 17	100 %	149	100 %
K26A	100	6.6 ± 0.1	7 %	134 ± 6.9	23 %	49	33 %
D90A	100	90.9 ± 3.2	103 %	488 ± 47	83 %	186	125 %
DM	150	160 ± 5.6	100 %	1157 ± 84	100 %	138	100 %
K26A	150	8.1 ± 0.2	5 %	156 ± 14	13 %	52	38 %
D90A	150	136 ± 4.0	85 %	723 ± 51	62 %	189	137 %

PfGrx	[GSH] (µM)	$k_{cat}^{app}{}_{(GSSCys)}$ [a] (s^{-1})		$K_m^{app}{}_{(GSSCys)}$ [a] (µM)		$k_{cat}^{app}/K_m^{app}{}_{(GSSCys)}$ (mM^{-1}s^{-1})	
DM	300	33.3 ± 0.4	100 %	25.3 ± 1.2	100 %	1399	100 %
K26A	300	10.3 ± 0.9	31 %	139 ± 21	549 %	74	5 %
D90A	300	50.4 ± 1.0	151 %	45.6 ± 2.8	180 %	1105	79 %
DM	500	56.4 ± 0.6	100 %	42.8 ± 1.4	100 %	1351	100 %
K26A	500	17.2 ± 2.4	30 %	235 ± 48	549 %	73	5 %
D90A	500	84.9 ± 5.1	151 %	84.6 ± 12	198 %	1003	74 %
DM	1000	98.2 ± 2.5	100 %	73.1 ± 4.7	100 %	1316	100 %
K26A	1000	25.2 ± 4.5	26 %	361 ± 86	494 %	70	5 %
D90A	1000	168 ± 7.9	171 %	167 ± 14	228 %	1004	76 %
DM	2000	112 ± 2.4	100 %	82.9 ± 4.0	100 %	1311	100 %
K26A	2000	36.1 ± 7.4	32 %	516 ± 130	622 %	70	5 %
D90A	2000	265 ± 15	237 %	260 ± 22	314 %	1019	78 %

[a] Mean±SD from Michaelis-Menten plots of at least two independent protein purifications. Percentages are relative to PfGrx$^{C32S/C88S}$. P-values are listed in Supplementary Table 11.

Supplementary Table 7. Selected k_{cat}^{app} and K_m^{app} values from HEDS assays with PfGrx$^{C32S/C88S}$ and the mutants K26A and D90A from Supplementary Fig. 14.

PfGrx	[HEDS] (mM)	$k_{cat}^{app}{}_{(GSH)}$ [a] (s^{-1})		$K_m^{app}{}_{(GSH)}$ [a] (mM)		$k_{cat}^{app}/K_m^{app}{}_{(GSH)}$ ($mM^{-1}s^{-1}$)	
DM	0.18	37.1 ± 2.0	100 %	1.84 ± 0.20	100 %	20.2	100 %
K26A	0.18	2.3 ± 0.1	6 %	1.96 ± 0.24	107 %	1.2	6 %
D90A	0.18	19.8 ± 1.5	53 %	1.37 ± 0.19	74 %	14.5	72 %
DM	0.37	66.7 ± 3.0	100 %	1.86 ± 0.17	100 %	35.9	100 %
K26A	0.37	4.5 ± 0.3	7 %	2.12 ± 0.23	114 %	2.1	6 %
D90A	0.37	36.7 ± 3.5	55 %	1.51 ± 0.26	81 %	24.3	68 %
DM	0.55	96.0 ± 2.1	100 %	2.08 ± 0.09	100 %	46.2	100 %
K26A	0.55	6.5 ± 0.3	7 %	2.38 ± 0.19	114 %	2.7	6 %
D90A	0.55	65.6 ± 8.8	68 %	2.27 ± 0.49	109 %	28.8	62 %
DM	0.74	134 ± 9.1	100 %	2.86 ± 0.31	100 %	46.9	100 %
K26A	0.74	7.2 ± 0.3	5 %	1.72 ± 0.17	60 %	4.2	9 %
D90A	0.74	101 ± 10	75 %	3.29 ± 0.48	115 %	30.8	66 %

PfGrx	[GSH] (mM)	$k_{cat}^{app}{}_{(HEDS)}$ [a] (s^{-1})		$K_m^{app}{}_{(HEDS)}$ [a] (mM)		$k_{cat}^{app}/K_m^{app}{}_{(HEDS)}$ ($mM^{-1}s^{-1}$)	
DM	0.25	20.1 ± 1.0	100 %	0.51 ± 0.05	100 %	39.3	100 %
K26A	0.25	2.1 ± 1.1	11 %	1.30 ± 0.86	253 %	1.7	4 %
D90A	0.25	13.6 ± 0.7	68 %	0.68 ± 0.06	133 %	20.0	51 %
DM	0.5	40.6 ± 0.7	100 %	0.57 ± 0.02	100 %	71.2	100 %
K26A	0.5	3.7 ± 1.5	9 %	1.16 ± 0.91	203 %	3.2	4 %
D90A	0.5	23.6 ± 0.6	58 %	0.61 ± 0.03	108 %	38.4	54 %
DM	1.0	78.2 ± 2.0	100 %	0.75 ± 0.03	100 %	105	100 %
K26A	1.0	8.5 ± 2.4	11 %	1.80 ± 0.98	241 %	4.7	4 %
D90A	1.0	58.3 ± 2.6	75 %	1.02 ± 0.07	137 %	57.1	55 %
DM	2.0	130 ± 7.4	100 %	0.76 ± 0.08	100 %	170	100 %
K26A	2.0	14.8 ± 3.9	11 %	2.06 ± 0.80	270 %	7.2	4 %
D90A	2.0	129 ± 18	99 %	1.83 ± 0.35	241 %	70.2	41 %

[a] Mean±SD from Michaelis-Menten plots of at least three independent protein purifications. Percentages are relative to PfGrx$^{C32S/C88S}$ (DM). P-values are listed in Supplementary Table 11.

Supplementary Table 8. Comparison of estimated true k_{cat} and K_m values and Dalziel coefficients of $PfGrx^{C32S/C88S}$ and the mutants K26A and D90A in the GSSCys and HEDS assay.

GSSCys assay

**PfGrx**	**$1/\Phi_1$** [a] ($M^{-1}s^{-1}$)	**$1/\Phi_2$** [a] ($M^{-1}s^{-1}$)	**k_{cat}** [a] (s^{-1})	**$K_{m\ (GSH)}$** [a] (mM)	**$K_{m\ (GSSCys)}$** [a] (mM)
DM	1.3×10^6	1.2×10^5	∞	∞	∞
K26A	6.9×10^4	5.0×10^4	34 - ∞	0.7 - ∞	1.2 - ∞
DM	1.4×10^6	1.2×10^5	289 - ∞	2.2 - ∞	0.4 - ∞
D90A	1.0×10^6	1.7×10^5	∞	∞	∞

HEDS assay

**PfGrx**	**$1/\Phi_1$** [b] ($M^{-1}s^{-1}$)	**$1/\Phi_2$** [b] ($M^{-1}s^{-1}$)	**k_{cat}** [b] (s^{-1})	**$K_{m\ (GSH)}$** [c] (mM)	**$K_{m\ (HEDS)}$** [c] (mM)
DM	2.2×10^5	8.3×10^4	∞	∞	∞
K26A	1.4×10^4	3.1×10^4	22 - ∞	2.5 - ∞	2.1 - ∞
DM	2.7×10^5	7.1×10^4	∞	∞	∞
D90A	1.0×10^5	5.3×10^4	∞	∞	∞

[a] Estimated k_{cat}, K_m values and Dalziel coefficients were obtained from Supplementary Figs. 12.
[b] Estimated k_{cat} values and Dalziel coefficients were obtained from Supplementary Fig. 15.
[c] Estimated K_m values were obtained from Supplementary Figs. 14 and 15.

Supplementary Table 9. List of ScGrx7 mutagenesis primers.

Primer	**Sequence** (codon and mutation highlighted)
ScGrx7/K105R/s	5'- CATGATTGTATTTAGC*AGG*ACTGGCTGCCCATATAG -3'
ScGrx7/K105R/as	5'- CTATATGGGCAGCCAGT*CCT*GCTAAATACAATCATG -3'
ScGrx7/K105A/s	5'- CATGATTGTATTTAGC*GCG*ACTGGCTGCCCATATAG -3'
ScGrx7/K105A/as	5'- CTATATGGGCAGCCAGT*CGC*GCTAAATACAATCATG -3'
ScGrx7/K105Y/s	5'- CATGATTGTATTTAGC*TAT*ACTGGCTGCCCATATAG -3'
ScGrx7/K105Y/as	5'- CTATATGGGCAGCCAGT*ATA*GCTAAATACAATCATG -3'
ScGrx7/K105E/s	5'- CATGATTGTATTTAGC*GAG*ACTGGCTGCCCATATAG -3'
ScGrx7/K105E/as	5'- CTATATGGGCAGCCAGT*CTC*GCTAAATACAATCATG -3'
ScGrx7/E170D/s	5'- CCAGAGGTGGTTATACT*GAT*ATAGCAGAGTTACATAA -3'
ScGrx7/E170D/as	5'- TTATGTAACTCTGCTAT*ATC*AGTATAACCACCTCTGG -3'
ScGrx7/E170A/s	5'- CCAGAGGTGGTTATACT*GCG*ATAGCAGAGTTACATAA -3'
ScGrx7/E170A/as	5'- TTATGTAACTCTGCTAT*CGC*AGTATAACCACCTCTGG -3'
ScGrx7/E170K/s	5'- CCAGAGGTGGTTATACT*AAG*ATAGCAGAGTTACATAA -3'
ScGrx7/E170K/as	5'- TTATGTAACTCTGCTAT*CTT*AGTATAACCACCTCTGG -3'
ScGrx7/Y110A/s	5'- GCAAGACTGGCTGCCCA*GCT*AGCAAAAAACTGAAAGC-3'
ScGrx7/Y110A/as	5'- GCTTTCAGTTTTTTGCT*AGC*TGGGCAGCCAGTCTTGC -3'

Supplementary Table 10. List of PfGrx mutagenesis primers.

Primer	**Sequence** (codon and mutation highlighted)
PfGrx/K26A/s	5'- GAACATCATTGCTGTATTTGCA*GCA*ACGGAATGCCCATATAG -3'
PfGrx/K26A/as	5'- CTATATGGGCATTCCGT*TGC*TGCAAATACAGCAATGATGTTC -3'
PfGrx/D90A/s	5'- CGTTGTCGGCGGAAGTGAT*GCT*TTAGTTAAAGAAAATGATG -3'
PfGrx/D90A/as	5'- CATCATTTTCTTTAACTAA*AGC*ATCACTTCCGCCGACAACG -3'

Supplementary Table 11. Statistical analysis of k_{cat}^{app} and K_m^{app} values from the indicated measurements of ScGrx7 and PfGrx. Two-tailed P-values from pairwise t-tests were calculated in Sigmaplot 12.5 (P > 0.05 : ns; P ≤ 0.05 : *; P ≤ 0.01 : **, P ≤ 0.001 : ***).

A) GSSCys assay ScGrx7 K105X (Fig. 2, Supplementary Fig. 2 and Supplementary Table 2)

A1.1) $k_{cat}^{app}{}_{(GSSCys)}$ @ 50 µM GSH

Comparison		P-value	
WT	→ K105R	0.81	ns
WT	→ K105A	0.006	**
WT	→ K105Y	0.002	**
WT	→ K105E	<0.001	***
K105R	→ K105A	0.032	*
K105R	→ K105Y	0.021	*
K105R	→ K105E	0.014	*
K105A	→ K105Y	0.107	ns
K105A	→ K105E	0.019	*
K105Y	→ K105E	0.015	*

A1.2) $k_{cat}^{app}{}_{(GSSCys)}$ @ 100 µM GSH

Comparison		P-value	
WT	→ K105R	0.333	ns
WT	→ K105A	0.002	**
WT	→ K105Y	<0.001	***
WT	→ K105E	<0.001	***
K105R	→ K105A	0.003	**
K105R	→ K105Y	0.002	**
K105R	→ K105E	0.001	***
K105A	→ K105Y	0.184	ns
K105A	→ K105E	0.013	*
K105Y	→ K105E	0.001	***

A1.3) $k_{cat}^{app}{}_{(GSSCys)}$ @ 200 µM GSH

Comparison		P-value	
WT	→ K105R	0.052	ns
WT	→ K105A	0.001	***
WT	→ K105Y	0.002	**
WT	→ K105E	<0.001	***
K105R	→ K105A	0.012	*
K105R	→ K105Y	0.011	*
K105R	→ K105E	0.003	**
K105A	→ K105Y	0.192	ns
K105A	→ K105E	<0.001	***
K105Y	→ K105E	0.003	**

A1.4) $k_{cat}^{app}{}_{(GSSCys)}$ @ 300 µM GSH

Comparison		P-value	
WT	→ K105R	0.809	ns
WT	→ K105A	0.016	*
WT	→ K105Y	0.002	**
WT	→ K105E	<0.001	***
K105R	→ K105A	0.055	ns
K105R	→ K105Y	0.026	*
K105R	→ K105E	0.020	*
K105A	→ K105Y	0.121	ns
K105A	→ K105E	0.014	*
K105Y	→ K105E	0.013	*

A2.1) $k_{cat}^{app}{}_{(GSH)}$ @ 25 µM GSSCys

Comparison		P-value	
WT	→ K105R	<0.001	***
WT	→ K105A	<0.001	***
WT	→ K105Y	<0.001	***
WT	→ K105E	<0.001	***
K105R	→ K105A	<0.001	***
K105R	→ K105Y	<0.001	***
K105R	→ K105E	<0.001	***
K105A	→ K105Y	<0.001	***
K105A	→ K105E	<0.001	***
K105Y	→ K105E	<0.001	***

A2.2) $k_{cat}^{app}{}_{(GSH)}$ @ 50 µM GSSCys

Comparison		P-value	
WT	→ K105R	<0.001	***
WT	→ K105A	<0.001	***
WT	→ K105Y	<0.001	***
WT	→ K105E	<0.001	***
K105R	→ K105A	<0.001	***
K105R	→ K105Y	<0.001	***
K105R	→ K105E	<0.001	***
K105A	→ K105Y	<0.001	***
K105A	→ K105E	<0.001	***
K105Y	→ K105E	<0.001	***

A2.3) $k_{cat}^{app}{}_{(GSH)}$ @ 100 µM GSSCys

Comparison		P-value	
WT	→ K105R	<0.001	***
WT	→ K105A	<0.001	***
WT	→ K105Y	<0.001	***
WT	→ K105E	<0.001	***
K105R	→ K105A	<0.001	***
K105R	→ K105Y	<0.001	***
K105R	→ K105E	<0.001	***
K105A	→ K105Y	<0.001	***
K105A	→ K105E	<0.001	***
K105Y	→ K105E	<0.001	***

A2.4) $k_{cat}^{app}{}_{(GSH)}$ @ 150 µM GSSCys

Comparison		P-value	
WT	→ K105R	<0.001	***
WT	→ K105A	<0.001	***
WT	→ K105Y	<0.001	***
WT	→ K105E	<0.001	***
K105R	→ K105A	<0.001	***
K105R	→ K105Y	<0.001	***
K105R	→ K105E	<0.001	***
K105A	→ K105Y	<0.001	***
K105A	→ K105E	<0.001	***
K105Y	→ K105E	<0.001	***

A3.1) $K_m^{app}{}_{(GSSCys)}$ @ 50 µM GSH

Comparison		P-value	
WT	→ K105R	0.030	*
WT	→ K105A	0.039	*
WT	→ K105Y	0.026	*
WT	→ K105E	0.003	**
K105R	→ K105A	0.430	ns
K105R	→ K105Y	0.335	ns
K105R	→ K105E	0.131	ns
K105A	→ K105Y	0.864	ns
K105A	→ K105E	0.306	ns
K105Y	→ K105E	0.287	ns

A4.1) $K_m^{app}{}_{(GSH)}$ @ 25 µM GSSCys

Comparison		P-value	
WT	→ K105R	<0.001	***
WT	→ K105A	0.001	***
WT	→ K105Y	<0.001	***
WT	→ K105E	<0.001	***
K105R	→ K105A	0.343	ns
K105R	→ K105Y	0.997	ns
K105R	→ K105E	0.114	ns
K105A	→ K105Y	0.414	ns
K105A	→ K105E	0.480	ns
K105Y	→ K105E	0.171	ns

A3.2) $K_m^{app}{}_{(GSSCys)}$ @ 100 µM GSH

Comparison		P-value	
WT	→ K105R	0.002	**
WT	→ K105A	0.022	*
WT	→ K105Y	<0.001	***
WT	→ K105E	0.012	*
K105R	→ K105A	0.045	*
K105R	→ K105Y	0.077	ns
K105R	→ K105E	0.018	*
K105A	→ K105Y	0.097	ns
K105A	→ K105E	0.543	ns
K105Y	→ K105E	0.031	*

A4.2) $K_m^{app}{}_{(GSH)}$ @ 50 µM GSSCys

Comparison		P-value	
WT	→ K105R	<0.001	***
WT	→ K105A	<0.001	***
WT	→ K105Y	<0.001	***
WT	→ K105E	<0.001	***
K105R	→ K105A	0.102	ns
K105R	→ K105Y	0.015	*
K105R	→ K105E	0.180	ns
K105A	→ K105Y	0.013	*
K105A	→ K105E	0.045	*
K105Y	→ K105E	0.013	*

A3.3) $K_m^{app}{}_{(GSSCys)}$ @ 200 µM GSH

Comparison		P-value	
WT	→ K105R	0.040	*
WT	→ K105A	0.003	**
WT	→ K105Y	0.034	*
WT	→ K105E	0.020	*
K105R	→ K105A	0.637	ns
K105R	→ K105Y	0.862	ns
K105R	→ K105E	0.116	ns
K105A	→ K105Y	0.757	ns
K105A	→ K105E	0.049	*
K105Y	→ K105E	0.153	ns

A4.3) $K_m^{app}{}_{(GSH)}$ @ 100 µM GSSCys

Comparison		P-value	
WT	→ K105R	0.003	**
WT	→ K105A	0.003	**
WT	→ K105Y	0.003	**
WT	→ K105E	0.003	**
K105R	→ K105A	0.017	*
K105R	→ K105Y	0.201	ns
K105R	→ K105E	0.234	ns
K105A	→ K105Y	0.012	*
K105A	→ K105E	0.028	*
K105Y	→ K105E	0.076	ns

A3.4) $K_m^{app}{}_{(GSSCys)}$ @ 300 µM GSH

Comparison		P-value	
WT	→ K105R	0.007	**
WT	→ K105A	0.015	*
WT	→ K105Y	0.018	*
WT	→ K105E	0.002	**
K105R	→ K105A	0.257	ns
K105R	→ K105Y	0.136	ns
K105R	→ K105E	0.082	ns
K105A	→ K105Y	0.308	ns
K105A	→ K105E	0.143	ns
K105Y	→ K105E	0.297	ns

A4.4) $K_m^{app}{}_{(GSH)}$ @ 150 µM GSSCys

Comparison		P-value	
WT	→ K105R	0.001	***
WT	→ K105A	0.001	***
WT	→ K105Y	0.001	***
WT	→ K105E	0.001	***
K105R	→ K105A	0.032	*
K105R	→ K105Y	0.274	ns
K105R	→ K105E	0.048	*
K105A	→ K105Y	0.033	*
K105A	→ K105E	0.498	ns
K105Y	→ K105E	0.045	*

B) HEDS assay ScGrx7 K105X (Fig. 3, Supplementary Fig. 4 and Supplementary Table 3)

B1.1) $k_{cat}^{app}{}_{(HEDS)}$ @ 300 µM GSH

Comparison		P-value	
WT	→ K105R	0.043	*
WT	→ K105A	0.030	*
WT	→ K105Y	0.029	*
WT	→ K105E	0.027	*
K105R	→ K105A	0.002	**
K105R	→ K105Y	0.002	**
K105R	→ K105E	0.001	***
K105A	→ K105Y	0.255	ns
K105A	→ K105E	0.022	*
K105Y	→ K105E	0.062	ns

B1.2) $k_{cat}^{app}{}_{(HEDS)}$ @ 500 µM GSH

Comparison		P-value	
WT	→ K105R	<0.001	***
WT	→ K105A	<0.001	***
WT	→ K105Y	<0.001	***
WT	→ K105E	<0.001	***
K105R	→ K105A	0.064	ns
K105R	→ K105Y	0.038	*
K105R	→ K105E	0.025	*
K105A	→ K105Y	0.226	ns
K105A	→ K105E	0.048	*
K105Y	→ K105E	0.045	*

B1.3) $k_{cat}^{app}{}_{(HEDS)}$ @ 1000 µM GSH

Comparison		P-value	
WT	→ K105R	<0.001	***
WT	→ K105A	<0.001	***
WT	→ K105Y	<0.001	***
WT	→ K105E	<0.001	***
K105R	→ K105A	0.011	*
K105R	→ K105Y	0.004	**
K105R	→ K105E	0.001	***
K105A	→ K105Y	0.047	*
K105A	→ K105E	<0.001	***
K105Y	→ K105E	0.006	**

B1.4) $k_{cat}^{app}{}_{(HEDS)}$ @ 1500 µM GSH

Comparison		P-value	
WT	→ K105R	0.003	**
WT	→ K105A	0.003	**
WT	→ K105Y	0.002	**
WT	→ K105E	0.002	**
K105R	→ K105A	0.038	*
K105R	→ K105Y	<0.001	***
K105R	→ K105E	<0.001	***
K105A	→ K105Y	0.032	*
K105A	→ K105E	0.012	*
K105Y	→ K105E	0.001	**

B2.1) $k_{cat}^{app}{}_{(GSH)}$ @ 0.18 mM HEDS

Comparison		P-value	
WT	→ K105R	<0.001	***
WT	→ K105A	<0.001	***
WT	→ K105Y	<0.001	***
WT	→ K105E	<0.001	***
K105R	→ K105A	<0.001	***
K105R	→ K105Y	<0.001	***
K105R	→ K105E	<0.001	***
K105A	→ K105Y	0.169	ns
K105A	→ K105E	<0.001	***
K105Y	→ K105E	<0.001	***

B2.2) $k_{cat}^{app}{}_{(GSH)}$ @ 0.37 mM HEDS

Comparison		P-value	
WT	→ K105R	<0.001	***
WT	→ K105A	<0.001	***
WT	→ K105Y	<0.001	***
WT	→ K105E	<0.001	***
K105R	→ K105A	<0.001	***
K105R	→ K105Y	<0.001	***
K105R	→ K105E	<0.001	***
K105A	→ K105Y	<0.001	***
K105A	→ K105E	<0.001	***
K105Y	→ K105E	<0.001	***

B2.3) $k_{cat}^{app}{}_{(GSH)}$ @ 0.55 mM HEDS

Comparison		P-value	
WT	→ K105R	<0.001	***
WT	→ K105A	<0.001	***
WT	→ K105Y	<0.001	***
WT	→ K105E	<0.001	***
K105R	→ K105A	<0.001	***
K105R	→ K105Y	<0.001	***
K105R	→ K105E	<0.001	***
K105A	→ K105Y	<0.001	***
K105A	→ K105E	<0.001	***
K105Y	→ K105E	<0.001	***

B2.4) $k_{cat}^{app}{}_{(GSH)}$ @ 0.74 mM HEDS

Comparison		P-value	
WT	→ K105R	<0.001	***
WT	→ K105A	<0.001	***
WT	→ K105Y	<0.001	***
WT	→ K105E	<0.001	***
K105R	→ K105A	<0.001	***
K105R	→ K105Y	<0.001	***
K105R	→ K105E	<0.001	***
K105A	→ K105Y	<0.001	***
K105A	→ K105E	<0.001	***
K105Y	→ K105E	<0.001	***

B3.1) $K_m^{app}{}_{(HEDS)}$ @ 300 µM GSH

Comparison		P-value	
WT	→ K105R	0.063	ns
WT	→ K105A	0.981	ns
WT	→ K105Y	0.896	ns
WT	→ K105E	0.262	ns
K105R	→ K105A	0.028	*
K105R	→ K105Y	0.046	*
K105R	→ K105E	0.554	ns
K105A	→ K105Y	0.768	ns
K105A	→ K105E	0.242	ns
K105Y	→ K105E	0.261	ns

B3.2) $K_m^{app}{}_{(HEDS)}$ @ 500 µM GSH

Comparison		P-value	
WT	→ K105R	0.229	ns
WT	→ K105A	0.534	ns
WT	→ K105Y	0.633	ns
WT	→ K105E	0.300	ns
K105R	→ K105A	0.450	ns
K105R	→ K105Y	0.265	ns
K105R	→ K105E	0.622	ns
K105A	→ K105Y	0.648	ns
K105A	→ K105E	0.387	ns
K105Y	→ K105E	0.318	ns

B3.3) $K_m^{app}{}_{(HEDS)}$ @ 1000 µM GSH

Comparison		P-value	
WT	→ K105R	0.082	ns
WT	→ K105A	0.043	*
WT	→ K105Y	0.386	ns
WT	→ K105E	0.037	*
K105R	→ K105A	0.896	ns
K105R	→ K105Y	0.345	ns
K105R	→ K105E	0.668	ns
K105A	→ K105Y	0.258	ns
K105A	→ K105E	0.735	ns
K105Y	→ K105E	0.188	ns

B3.4) $K_m^{app}{}_{(HEDS)}$ @ 1500 µM GSH

Comparison		P-value	
WT	→ K105R	0.242	ns
WT	→ K105A	0.928	ns
WT	→ K105Y	0.046	*
WT	→ K105E	0.622	ns
K105R	→ K105A	0.319	ns
K105R	→ K105Y	0.068	ns
K105R	→ K105E	0.147	ns
K105A	→ K105Y	0.087	ns
K105A	→ K105E	0.733	ns
K105Y	→ K105E	0.038	*

B4.1) $K_m^{app}{}_{(GSH)}$ @ 0.18 mM HEDS

Comparison		P-value	
WT	→ K105R	0.028	*
WT	→ K105A	0.936	ns
WT	→ K105Y	0.151	ns
WT	→ K105E	0.079	ns
K105R	→ K105A	0.002	**
K105R	→ K105Y	0.090	ns
K105R	→ K105E	0.323	ns
K105A	→ K105Y	0.035	*
K105A	→ K105E	0.016	*
K105Y	→ K105E	0.452	ns

B4.2) $K_m^{app}{}_{(GSH)}$ @ 0.37 mM HEDS

Comparison		P-value	
WT	→ K105R	0.012	*
WT	→ K105A	0.160	ns
WT	→ K105Y	0.055	ns
WT	→ K105E	0.207	ns
K105R	→ K105A	0.002	**
K105R	→ K105Y	0.011	*
K105R	→ K105E	0.005	**
K105A	→ K105Y	0.045	*
K105A	→ K105E	0.801	ns
K105Y	→ K105E	0.086	ns

B4.3) $K_m^{app}{}_{(GSH)}$ @ 0.55 mM HEDS

Comparison		P-value	
WT	→ K105R	0.011	*
WT	→ K105A	0.220	ns
WT	→ K105Y	0.037	*
WT	→ K105E	0.045	*
K105R	→ K105A	0.030	*
K105R	→ K105Y	0.166	ns
K105R	→ K105E	0.891	ns
K105A	→ K105Y	0.158	ns
K105A	→ K105E	0.126	ns
K105Y	→ K105E	0.437	ns

B4.4) $K_m^{app}{}_{(GSH)}$ @ 0.74 mM HEDS

Comparison		P-value	
WT	→ K105R	0.449	ns
WT	→ K105A	0.074	ns
WT	→ K105Y	0.334	ns
WT	→ K105E	0.197	ns
K105R	→ K105A	0.385	ns
K105R	→ K105Y	0.893	ns
K105R	→ K105E	0.622	ns
K105A	→ K105Y	0.097	ns
K105A	→ K105E	0.717	ns
K105Y	→ K105E	0.401	ns

C) GSSCys assay ScGrx7 E170X (Fig. 4, Supplementary Fig. 6 and Supplementary Table 4)

C1.1) $k_{cat}^{app}{}_{(GSSCys)}$ @ 100 µM GSH

Comparison		P-value	
WT	→ E170D	0.004	**
WT	→ E170A	0.010	**
WT	→ E170K	0.007	**
E170D	→ E170A	0.002	**
E170D	→ E170K	0.001	**
E170A	→ E170K	0.0953	ns

C1.2) $k_{cat}^{app}{}_{(GSSCys)}$ @ 200 µM GSH

Comparison		P-value	
WT	→ E170D	0.011	*
WT	→ E170A	0.063	ns
WT	→ E170K	0.022	*
E170D	→ E170A	0.003	**
E170D	→ E170K	0.003	**
E170A	→ E170K	0.061	ns

C1.3) $k_{cat}^{app}{}_{(GSSCys)}$ @ 300 µM GSH

Comparison		P-value	
WT	→ E170D	0.036	*
WT	→ E170A	0.019	*
WT	→ E170K	0.005	**
E170D	→ E170A	0.010	**
E170D	→ E170K	0.003	**
E170A	→ E170K	0.330	ns

C1.4) $k_{cat}^{app}{}_{(GSSCys)}$ @ 1000 µM GSH

Comparison		P-value	
WT	→ E170D	0.137	ns
WT	→ E170A	0.339	ns
WT	→ E170K	0.107	ns
E170D	→ E170A	0.099	ns
E170D	→ E170K	0.050	*
E170A	→ E170K	0.608	ns

C2.1) $k_{cat}^{app}{}_{(GSH)}$ @ 25 µM GSSCys

Comparison		P-value	
WT	→ E170D	0.294	ns
WT	→ E170A	0.002	**
WT	→ E170K	0.001	***
E170D	→ E170A	0.005	**
E170D	→ E170K	0.003	**
E170A	→ E170K	0.026	*

C2.2) $k_{cat}^{app}{}_{(GSH)}$ @ 50 µM GSSCys

Comparison		P-value	
WT	→ E170D	0.323	ns
WT	→ E170A	0.003	**
WT	→ E170K	0.002	**
E170D	→ E170A	0.006	**
E170D	→ E170K	0.004	**
E170A	→ E170K	0.419	ns

C2.3) $k_{cat}^{app}{}_{(GSH)}$ @ 100 µM GSSCys

Comparison		P-value	
WT	→ E170D	0.245	ns
WT	→ E170A	0.003	**
WT	→ E170K	0.002	**
E170D	→ E170A	0.004	**
E170D	→ E170K	0.001	**
E170A	→ E170K	0.234	ns

C2.4) $k_{cat}^{app}{}_{(GSH)}$ @ 150 µM GSSCys

Comparison		P-value	
WT	→ E170D	0.658	ns
WT	→ E170A	0.007	**
WT	→ E170K	0.006	**
E170D	→ E170A	0.004	**
E170D	→ E170K	0.002	**
E170A	→ E170K	0.190	ns

C3.1) $K_m^{app}{}_{(GSSCys)}$ @ 100 µM GSH

Comparison		P-value	
WT	→ E170D	0.369	ns
WT	→ E170A	0.005	**
WT	→ E170K	0.005	**
E170D	→ E170A	0.005	**
E170D	→ E170K	0.005	**
E170A	→ E170K	0.174	ns

C3.2) $K_m^{app}{}_{(GSSCys)}$ @ 200 µM GSH

Comparison		P-value	
WT	→ E170D	0.533	ns
WT	→ E170A	0.008	**
WT	→ E170K	0.006	**
E170D	→ E170A	0.005	**
E170D	→ E170K	0.004	**
E170A	→ E170K	0.080	ns

C3.3) $K_m^{app}{}_{(GSSCys)}$ @ 300 µM GSH

Comparison		P-value	
WT	→ E170D	0.869	ns
WT	→ E170A	0.009	**
WT	→ E170K	0.002	**
E170D	→ E170A	0.011	*
E170D	→ E170K	0.003	**
E170A	→ E170K	0.161	ns

C3.4) $K_m^{app}{}_{(GSSCys)}$ @ 1000 µM GSH

Comparison		P-value	
WT	→ E170D	0.756	ns
WT	→ E170A	0.010	**
WT	→ E170K	0.003	**
E170D	→ E170A	0.015	*
E170D	→ E170K	0.006	**
E170A	→ E170K	0.280	ns

C4.1) $K_m^{app}{}_{(GSH)}$ @ 25 µM GSSCys

Comparison		P-value	
WT	→ E170D	0.096	ns
WT	→ E170A	0.021	*
WT	→ E170K	0.009	**
E170D	→ E170A	0.019	*
E170D	→ E170K	0.012	*
E170A	→ E170K	0.061	ns

C4.2) $K_m^{app}{}_{(GSH)}$ @ 50 µM GSSCys

Comparison		P-value	
WT	→ E170D	0.116	ns
WT	→ E170A	0.018	*
WT	→ E170K	0.007	**
E170D	→ E170A	0.016	**
E170D	→ E170K	0.010	**
E170A	→ E170K	0.452	ns

C4.3) $K_m^{app}{}_{(GSH)}$ @ 100 µM GSSCys

Comparison		P-value	
WT	→ E170D	0.056	ns
WT	→ E170A	0.011	*
WT	→ E170K	0.005	**
E170D	→ E170A	0.006	**
E170D	→ E170K	0.003	**
E170A	→ E170K	0.383	ns

C4.4) $K_m^{app}{}_{(GSH)}$ @ 150 µM GSSCys

Comparison		P-value	
WT	→ E170D	0.136	ns
WT	→ E170A	0.017	*
WT	→ E170K	0.013	*
E170D	→ E170A	0.005	**
E170D	→ E170K	0.004	**
E170A	→ E170K	0.323	ns

D) HEDS assay ScGrx7 E170X (Fig. 5, Supplementary Fig. 8 and Supplementary Table 5)

D1.1) $k_{cat}^{app}{}_{(HEDS)}$ @ 300 µM GSH

Comparison		P-value	
WT	→ E170D	<0.001	***
WT	→ E170A	0.003	**
WT	→ E170K	0.021	*
E170D	→ E170A	0.106	ns
E170D	→ E170K	0.005	**
E170A	→ E170K	0.054	ns

D1.2) $k_{cat}^{app}{}_{(HEDS)}$ @ 500 µM GSH

Comparison		P-value	
WT	→ E170D	0.273	ns
WT	→ E170A	0.095	ns
WT	→ E170K	0.022	*
E170D	→ E170A	0.456	ns
E170D	→ E170K	0.159	ns
E170A	→ E170K	0.487	ns

D1.3) $k_{cat}^{app}{}_{(HEDS)}$ @ 1000 µM GSH

Comparison		P-value	
WT	→ E170D	0.373	ns
WT	→ E170A	<0.001	***
WT	→ E170K	0.001	***
E170D	→ E170A	<0.001	***
E170D	→ E170K	0.002	**
E170A	→ E170K	0.381	ns

D1.4) $k_{cat}^{app}{}_{(HEDS)}$ @ 1500 µM GSH

Comparison		P-value	
WT	→ E170D	0.163	ns
WT	→ E170A	0.002	**
WT	→ E170K	0.024	*
E170D	→ E170A	<0.001	***
E170D	→ E170K	0.042	*
E170A	→ E170K	0.022	*

D2.1) $k_{cat}^{app}{}_{(GSH)}$ @ 0.18 mM HEDS

Comparison		P-value	
WT	→ E170D	0.006	**
WT	→ E170A	<0.001	***
WT	→ E170K	<0.001	***
E170D	→ E170A	<0.001	***
E170D	→ E170K	<0.001	***
E170A	→ E170K	0.003	**

D2.2) $k_{cat}^{app}{}_{(GSH)}$ @ 0.37 mM HEDS

Comparison		P-value	
WT	→ E170D	0.030	*
WT	→ E170A	<0.001	***
WT	→ E170K	<0.001	***
E170D	→ E170A	<0.001	***
E170D	→ E170K	<0.001	***
E170A	→ E170K	0.005	**

D2.3) $k_{cat}^{app}{}_{(GSH)}$ @ 0.55 mM HEDS

Comparison		P-value	
WT	→ E170D	0.003	**
WT	→ E170A	<0.001	***
WT	→ E170K	<0.001	***
E170D	→ E170A	<0.001	***
E170D	→ E170K	<0.001	***
E170A	→ E170K	0.023	*

D2.4) $k_{cat}^{app}{}_{(GSH)}$ @ 0.74 mM HEDS

Comparison		P-value	
WT	→ E170D	0.008	**
WT	→ E170A	<0.001	***
WT	→ E170K	<0.001	***
E170D	→ E170A	<0.001	***
E170D	→ E170K	0.003	**
E170A	→ E170K	<0.001	***

D3.1) $K_m^{app}{}_{(HEDS)}$ @ 300 µM GSH

Comparison		P-value	
WT	→ E170D	0.001	**
WT	→ E170A	<0.001	***
WT	→ E170K	<0.001	***
E170D	→ E170A	<0.001	***
E170D	→ E170K	<0.001	***
E170A	→ E170K	0.571	ns

D3.2) $K_m^{app}{}_{(HEDS)}$ @ 500 µM GSH

Comparison		P-value	
WT	→ E170D	0.154	ns
WT	→ E170A	<0.001	***
WT	→ E170K	<0.001	***
E170D	→ E170A	<0.001	***
E170D	→ E170K	<0.001	***
E170A	→ E170K	0.003	**

D3.3) $K_m^{app}{}_{(HEDS)}$ @ 1000 µM GSH

Comparison		P-value	
WT	→ E170D	0.273	ns
WT	→ E170A	<0.001	***
WT	→ E170K	<0.001	***
E170D	→ E170A	<0.001	***
E170D	→ E170K	<0.001	***
E170A	→ E170K	0.012	*

D3.4) $K_m^{app}{}_{(HEDS)}$ @ 1500 µM GSH

Comparison		P-value	
WT	→ E170D	0.358	ns
WT	→ E170A	0.001	***
WT	→ E170K	0.001	***
E170D	→ E170A	<0.001	***
E170D	→ E170K	<0.001	***
E170A	→ E170K	0.505	ns

D4.1) $K_m^{app}{}_{(GSH)}$ @ 0.18 mM HEDS

Comparison		P-value	
WT	→ E170D	0.023	*
WT	→ E170A	0.053	ns
WT	→ E170K	0.078	ns
E170D	→ E170A	0.208	ns
E170D	→ E170K	0.073	ns
E170A	→ E170K	0.381	ns

D4.2) $K_m^{app}{}_{(GSH)}$ @ 0.37 mM HEDS

Comparison		P-value	
WT	→ E170D	0.331	ns
WT	→ E170A	0.171	ns
WT	→ E170K	0.141	ns
E170D	→ E170A	0.523	ns
E170D	→ E170K	0.538	ns
E170A	→ E170K	0.786	ns

D4.3) $K_m^{app}{}_{(GSH)}$ @ 0.55 mM HEDS

Comparison		P-value	
WT	→ E170D	0.069	ns
WT	→ E170A	0.049	*
WT	→ E170K	0.026	*
E170D	→ E170A	0.673	ns
E170D	→ E170K	0.296	ns
E170A	→ E170K	0.495	ns

D4.4) $K_m^{app}{}_{(GSH)}$ @ 0.74 mM HEDS

Comparison		P-value	
WT	→ E170D	0.100	ns
WT	→ E170A	0.022	*
WT	→ E170K	0.045	*
E170D	→ E170A	0.265	ns
E170D	→ E170K	0.914	ns
E170A	→ E170K	0.013	*

E) GSSCys assay PfGrx (Supplementary Figs. 11 and 13 and Supplementary Table 6)

E1.1) $k_{cat}^{app}{}_{(GSSCys)}$ @ 300 µM GSH

Comparison		P-value	
DM	→ K26A	<0.001	***
DM	→ D90A	<0.001	***
K26A	→ D90A	<0.001	***

E1.2) $k_{cat}^{app}{}_{(GSSCys)}$ @ 500 µM GSH

Comparison		P-value	
DM	→ K26A	<0.001	***
DM	→ D90A	<0.001	***
K26A	→ D90A	<0.001	***

E1.3) $k_{cat}^{app}{}_{(GSSCys)}$ @ 1000 µM GSH

Comparison		P-value	
DM	→ K26A	<0.001	***
DM	→ D90A	<0.001	***
K26A	→ D90A	<0.001	***

E1.4) $k_{cat}^{app}{}_{(GSSCys)}$ @ 2000 µM GSH

Comparison		P-value	
DM	→ K26A	<0.001	***
DM	→ D90A	<0.001	***
K26A	→ D90A	<0.001	***

E2.1) $k_{cat}^{app}{}_{(GSH)}$ @ 25 µM GSSCys

Comparison		P-value	
DM	→ K26A	<0.001	***
DM	→ D90A	<0.001	***
K26A	→ D90A	<0.001	***

E2.2) $k_{cat}^{app}{}_{(GSH)}$ @ 50 µM GSSCys

Comparison		P-value	
DM	→ K26A	<0.001	***
DM	→ D90A	0.002	**
K26A	→ D90A	<0.001	***

E2.3) $k_{cat}^{app}{}_{(GSH)}$ @ 100 µM GSSCys

Comparison		P-value	
DM	→ K26A	<0.001	***
DM	→ D90A	0.247	ns
K26A	→ D90A	<0.001	***

E2.4) $k_{cat}^{app}{}_{(GSH)}$ @ 150 µM GSSCys

Comparison		P-value	
DM	→ K26A	<0.001	***
DM	→ D90A	0.354	ns
K26A	→ D90A	<0.001	***

E3.1) $K_{m}^{app}{}_{(GSSCys)}$ @ 300 µM GSH

Comparison		P-value	
DM	→ K26A	<0.001	***
DM	→ D90A	<0.001	***
K26A	→ D90A	0.002	**

E3.2) $K_{m}^{app}{}_{(GSSCys)}$ @ 500 µM GSH

Comparison		P-value	
DM	→ K26A	0.002	**
DM	→ D90A	0.004	**
K26A	→ D90A	0.006	**

E3.3) $K_{m}^{app}{}_{(GSSCys)}$ @ 1000 µM GSH

Comparison		P-value	
DM	→ K26A	0.004	**
DM	→ D90A	<0.001	***
K26A	→ D90A	0.018	*

E3.4) $K_{m}^{app}{}_{(GSSCys)}$ @ 2000 µM GSH

Comparison		P-value	
DM	→ K26A	0.006	**
DM	→ D90A	<0.001	***
K26A	→ D90A	0.028	*

E4.1) $K_{m}^{app}{}_{(GSH)}$ @ 25 µM GSSCys

Comparison		P-value	
DM	→ K26A	<0.001	***
DM	→ D90A	0.002	**
K26A	→ D90A	0.001	***

E4.2) $K_{m}^{app}{}_{(GSH)}$ @ 50 µM GSSCys

Comparison		P-value	
DM	→ K26A	<0.001	***
DM	→ D90A	0.007	**
K26A	→ D90A	0.001	***

E4.3) $K_{m}^{app}{}_{(GSH)}$ @ 100 µM GSSCys

Comparison		P-value	
DM	→ K26A	<0.001	***
DM	→ D90A	0.232	ns
K26A	→ D90A	<0.001	***

E4.4) $K_{m}^{app}{}_{(GSH)}$ @ 150 µM GSSCys

Comparison		P-value	
DM	→ K26A	<0.001	***
DM	→ D90A	0.002	**
K26A	→ D90A	<0.001	***

F) HEDS assay PfGrx (Supplementary Figs. 14 and 16 and Supplementary Table 7)

F1.1) $k_{cat}^{app}{}_{(HEDS)}$ @ 250 µM GSH

Comparison		P-value	
DM	→ K26A	<0.001	***
DM	→ D90A	<0.001	***
K26A	→ D90A	<0.001	***

F1.2) $k_{cat}^{app}{}_{(HEDS)}$ @ 500 µM GSH

Comparison		P-value	
DM	→ K26A	<0.001	***
DM	→ D90A	<0.001	***
K26A	→ D90A	<0.001	***

F1.3) $k_{cat}^{app}{}_{(HEDS)}$ @ 1000 µM GSH

Comparison		P-value	
DM	→ K26A	<0.001	***
DM	→ D90A	<0.001	***
K26A	→ D90A	<0.001	***

F1.4) $k_{cat}^{app}{}_{(HEDS)}$ @ 2000 µM GSH

Comparison		P-value	
DM	→ K26A	<0.001	***
DM	→ D90A	0.933	ns
K26A	→ D90A	<0.001	***

F2.1) $k_{cat}^{app}{}_{(GSH)}$ @ 0.18 mM HEDS

Comparison		P-value	
DM	→ K26A	<0.001	***
DM	→ D90A	<0.001	***
K26A	→ D90A	<0.001	***

F2.2) $k_{cat}^{app}{}_{(GSH)}$ @ 0.37 mM HEDS

Comparison		P-value	
DM	→ K26A	<0.001	***
DM	→ D90A	<0.001	***
K26A	→ D90A	<0.001	***

F2.3) $k_{cat}^{app}{}_{(GSH)}$ @ 0.55 mM HEDS

Comparison		P-value	
DM	→ K26A	<0.001	***
DM	→ D90A	0.004	**
K26A	→ D90A	<0.001	***

F2.4) $k_{cat}^{app}{}_{(GSH)}$ @ 0.74 mM HEDS

Comparison		P-value	
DM	→ K26A	<0.001	***
DM	→ D90A	0.013	*
DM	→ K26A	<0.001	***

F3.1) $K_m^{app}{}_{(HEDS)}$ @ 250 µM GSH

Comparison		P-value	
DM	→ K26A	0.187	ns
DM	→ D90A	0.020	*
K26A	→ D90A	0.281	ns

F3.2) $K_m^{ap}{}_{(HEDS)}$ @ 500 µM GSH

Comparison		P-value	
DM	→ K26A	0.324	ns
DM	→ D90A	0.127	ns
K26A	→ D90A	0.354	ns

F3.3) $K_m^{app}{}_{(HEDS)}$ @ 1000 µM GSH

Comparison		P-value	
DM	→ K26A	0.137	ns
DM	→ D90A	0.004	**
K26A	→ D90A	0.241	ns

F3.4) $K_m^{app}{}_{(HEDS)}$ @ 2000 µM GSH

Comparison		P-value	
DM	→ K26A	0.049	*
DM	→ D90A	0.007	**
K26A	→ D90A	0.672	ns

F4.1) $K_m^{app}{}_{(GSH)}$ @ 0.18 mM HEDS

Comparison		P-value	
DM	→ K26A	0.542	ns
DM	→ D90A	0.042	*
K26A	→ D90A	0.029	*

F4.2) $K_m^{app}{}_{(GSH)}$ @ 0.37 mM HEDS

Comparison		P-value	
DM	→ K26A	0.190	ns
DM	→ D90A	0.015	*
K26A	→ D90A	0.038	*

F4.3) $K_m^{app}{}_{(GSH)}$ @ 0.55 mM HEDS

Comparison		P-value	
DM	→ K26A	0.069	ns
DM	→ D90A	0.118	ns
K26A	→ D90A	0.735	ns

F4.4) $K_m^{app}{}_{(GSH)}$ @ 0.74 mM HEDS

Comparison		P-value	
DM	→ K26A	0.005	**
DM	→ D90A	0.262	ns
K26A	→ D90A	0.006	**
